# Population-level mathematical modeling of antimicrobial resistance: a systematic review

**DOI:** 10.1186/s12916-019-1314-9

**Published:** 2019-04-24

**Authors:** Anna Maria Niewiadomska, Bamini Jayabalasingham, Jessica C. Seidman, Lander Willem, Bryan Grenfell, David Spiro, Cecile Viboud

**Affiliations:** 1 Division of International Epidemiology and Population Studies, Fogarty International Center, National Institutes of Health, Bethesda, USA; 2 Present Address: Elsevier Inc., 230 Park Ave, Suite B00, New York, NY 10169 USA; 3 University of Antwerp, Antwerp, Belgium; 4 Princeton University, Princeton, NJ USA

**Keywords:** Antimicrobial, Resistance, Mathematical, Computational, Models, Communicable diseases, Epidemiology, Transmission

## Abstract

**Background:**

Mathematical transmission models are increasingly used to guide public health interventions for infectious diseases, particularly in the context of emerging pathogens; however, the contribution of modeling to the growing issue of antimicrobial resistance (AMR) remains unclear. Here, we systematically evaluate publications on population-level transmission models of AMR over a recent period (2006–2016) to gauge the state of research and identify gaps warranting further work.

**Methods:**

We performed a systematic literature search of relevant databases to identify transmission studies of AMR in viral, bacterial, and parasitic disease systems. We analyzed the temporal, geographic, and subject matter trends, described the predominant medical and behavioral interventions studied, and identified central findings relating to key pathogens.

**Results:**

We identified 273 modeling studies; the majority of which (> 70%) focused on 5 infectious diseases (human immunodeficiency virus (HIV), influenza virus, *Plasmodium falciparum* (malaria), *Mycobacterium tuberculosis* (TB), and methicillin-resistant *Staphylococcus aureus* (MRSA)). AMR studies of influenza and nosocomial pathogens were mainly set in industrialized nations, while HIV, TB, and malaria studies were heavily skewed towards developing countries. The majority of articles focused on AMR exclusively in humans (89%), either in community (58%) or healthcare (27%) settings. Model systems were largely compartmental (76%) and deterministic (66%). Only 43% of models were calibrated against epidemiological data, and few were validated against out-of-sample datasets (14%). The interventions considered were primarily the impact of different drug regimens, hygiene and infection control measures, screening, and diagnostics, while few studies addressed de novo resistance, vaccination strategies, economic, or behavioral changes to reduce antibiotic use in humans and animals.

**Conclusions:**

The AMR modeling literature concentrates on disease systems where resistance has been long-established, while few studies pro-actively address recent rise in resistance in new pathogens or explore upstream strategies to reduce overall antibiotic consumption. Notable gaps include research on emerging resistance in Enterobacteriaceae and *Neisseria gonorrhoeae*; AMR transmission at the animal-human interface, particularly in agricultural and veterinary settings; transmission between hospitals and the community; the role of environmental factors in AMR transmission; and the potential of vaccines to combat AMR.

**Electronic supplementary material:**

The online version of this article (10.1186/s12916-019-1314-9) contains supplementary material, which is available to authorized users.

## Background

Antibiotics are commonly regarded as one the greatest discoveries of the twentieth century; however, antibiotic or antimicrobial resistance (AMR) is now a significant threat to global health. According to a World Health Organization (WHO) global report [1], healthcare-acquired infections (HCAI) with AMR pathogens such as methicillin-resistant *Staphyloccus aureus* are a serious problem in high- and middle-income countries where surveillance is well established. There are also indications that the prevalence of HCAIs in low-income countries may be greater than in higher-income regions, although epidemiological data are scarce [1, 2]. In addition to the threat posed by HCAIs, low-income countries need to contend with the emergence of drug resistance to long-standing pathogens, namely human immunodeficiency virus (HIV), tuberculosis (TB), and *Plasmodium* parasites (malaria) [1].

**Fig. 1:**
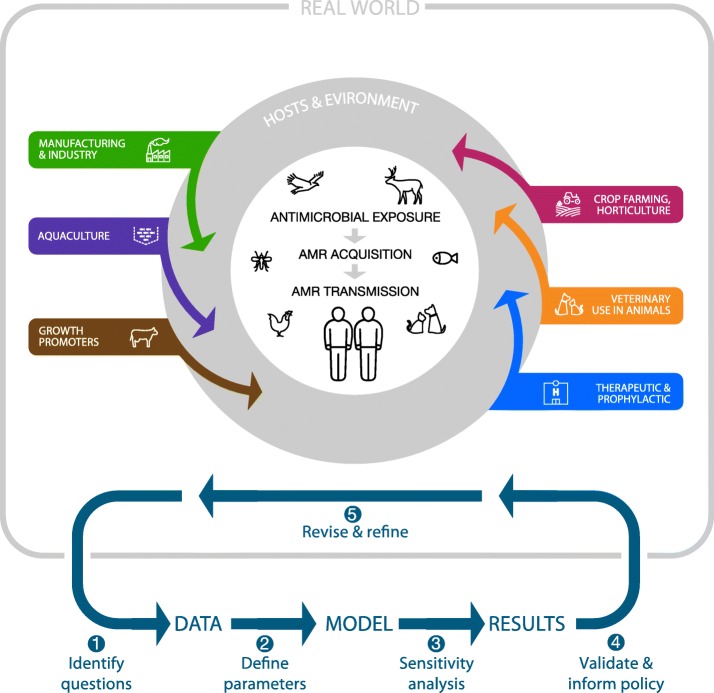
Sources of antimicrobial contamination, transmission of AMR, and development of mathematical models. Drivers of AMR as well as resistant pathogens themselves (antimicrobial, biocides, metals) may enter the environment through water (as effluent or through water sanitation systems) or soil (manure application or illegal dumping) from various sources including (i) medical therapeutic and prophylactic use in humans, (ii) veterinary use in companion or food animals, (iii) non-veterinary use in animals (growth promoters), (iv) direct or indirect use in horticulture and crop farming, (v) industrial scale prophylactic use in aquaculture, and (vi) pharmaceutical manufacturers themselves and various industrial applications. Resistant pathogens may then be transmitted to various living organisms through various routes including foodborne, waterborne, airborne, vectorborne, or direct contact. Zoonotic transmission is possible between humans and animals (domestic and wild). Transmission can be further intensified by insect vectors such as mosquitoes and flies, as well as human activity, such as global travel (tourism, migration) and food importation. The goal of mathematical modeling is to synthesize the data collected on AMR and design models to inform public health policy: step 1, identify key questions; step 2, extract or estimate disease parameters based on available data to build a model; step 3, assess model uncertainty/sensitivity; step 4, validate model results with an independent dataset and use to inform policy; and step 5, refine and revise model as needed with new data.

There is an abundance and diversity of sources of drug pressure favoring the emergence of AMR (Fig. 1) [1, 3, 4]. Antimicrobials produced by pharmaceutical manufacturers are distributed widely across a diverse array of industries and applications. Unnecessary or suboptimal use of antimicrobials in humans and animals for medical or prophylactic purposes can promote AMR. Antimicrobial use in animals for growth promotion and intensive crop farming also facilitate evolution of AMR organisms, which can then enter the food chain. Other nonmedical uses of antimicrobials include industrial manufacturing (anti-fouling paint, detergents, ethanol production, food preservations, etc.). Solid or liquid waste contaminated with either AMR organisms or antimicrobials from these many sources may then enter municipal sewer systems or waterways. Thus, antimicrobial release from pharmaceutical manufacturers and non-pharmaceutical industries, combined with human and agricultural use, can lead to contamination of the soil and water [3, 4].

Once primary antimicrobial resistance arises in an organism, it can spread through numerous routes, both within hosts (e.g., via plasmids or mobile elements that are common in bacterial genomes) and between hosts, or via contaminated environment (Fig. 1). There are multiple recognized routes of transmission of AMR pathogens from agricultural farms to humans [5, 6]. Soil and water can also transmit AMR organisms to humans, animals, and plants. Aerosol or airborne transmission is common for respiratory pathogens that may carry resistance such as influenza or tuberculosis, while vectors can facilitate the spread of resistant malaria or bacteria, facilitating rapid diffusion over vast geographic areas [7, 8]. While AMR cannot be realistically eradicated, it may be possible to slow down or reduce its occurrence through antimicrobial stewardship, namely, strategies designed to improve the appropriate use of antimicrobials.

Mathematical models are increasingly used to help understand and control infectious diseases, particularly to identify key parameters driving disease spread, assess the effect of potential interventions, and forecast the trajectory of epidemics [9]. The most impactful modeling studies typically involve close feedback between modelers, public health experts, and clinicians, to identify an actionable research question, design and calibrate a model against empirical data, perform sensitivity analyses, refine the model as more data become available, and eventually issue policy guidance (Fig. 1). Modeling AMR organisms can be particularly challenging compared to modeling sensitive pathogens for several reasons (see Box 1). In addition to crucial data gaps, modelers have to contend with issues of pathogen heterogeneity, fitness costs, co-infections, and competition, which are important features of resistance that remain poorly understood and quantified.

The contribution of mathematical modeling to the control of emerging infections is well established [9], and mathematical modeling can also be a powerful tool to guide policies to control AMR. Here, we undertake a systematic review to assess how population-level mathematical and computational modeling has been applied in the field of AMR over a period of 11 years (2006–2016). Previous reviews of AMR modeling were either completed some time ago [10, 11], only applied to a specific subset of AMR, such as HCAIs [12, 13], or focused on acquired resistance [14]. Our goals in this study were to (1) identify the predominant pathogens, populations, and interventions studied; (2) highlight recent advances in the field; (3) assess the influence of the research; and (4) identify gaps in both modeling of AMR and data availability.

## Methods

### Search strategy and selection criteria

**Fig. 2:**
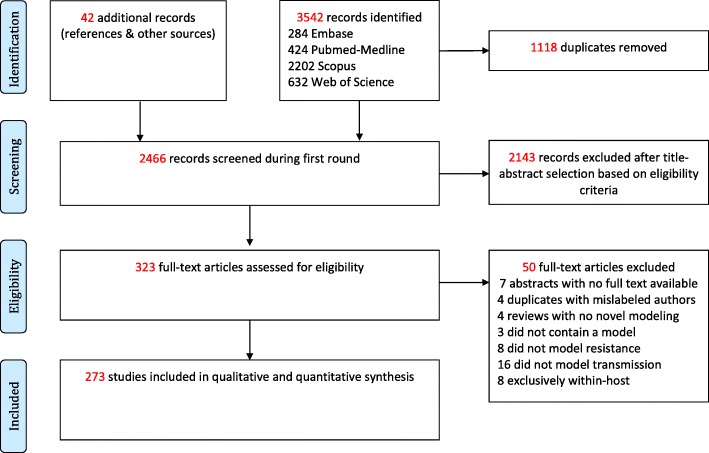
PRISMA flowchart outlining selection of studies included in the review.

We undertook a systematic search and review of publications relevant to the transmission modeling of AMR. Searches were carried out in PubMed-MEDLINE, Scopus, Web of Science, and Embase. Publications were limited by date (January 1, 2006–December 31, 2016) and journal type (original research and review articles only). Data extraction was initially carried out on November 15, 2016 and updated in January 2018. The search query included terms specific to transmission models, resistance issues, and individual pathogens known to acquire resistance (see Additional file 1 for details of the query). We removed duplicate publications and continued with the selection of relevant publications according to the inclusion/exclusion criteria listed below. A summary of the process is outlined in the PRISMA (Preferred Reporting Items for Systematic Reviews and Meta-analyses) diagram in Fig. 2 and in Additional file 2.

### Inclusion and exclusion criteria

We included any mathematical or computational models describing AMR in an infectious disease pathogen and considering transmission at the population level (i.e., publications on between-host transmission dynamics). We excluded within pathogen/host models of resistance (e.g., exclusively within-host models based on in vitro data), pharmacokinetic-pharmacodynamic models (i.e., pharmacological models focused on optimizing drug dosage that did not include a transmission component), molecular modeling studies (studies focused on molecular structure of chemical compounds), reviews that did not present original work, non-journal articles or reviews (poster or conference abstracts), and descriptive statistical models not incorporating mechanistic principles (such as models based on probability distributions, e.g., regression, clustering analysis).

### Selection and analysis of publications

An initial round of title and abstract screening was performed by AMN. Articles identified as potentially relevant were then reviewed by both AMN and CV, and the publication list for full-text analysis was agreed upon by consensus. Full texts for 313 articles were then retrieved, evaluated by AMN, and relevant data was extracted for further analysis (see below). For details on the number of articles excluded at each step, see Fig. 2.

### Data extraction

The following data were retrieved from articles: *disease system* (type: viral (V), bacterial (B), parasitic (P), fungal (F) or non-specific (NS)); *drug type*; *control measures* (pharmaceutical and non-pharmaceutical interventions, vaccines, behavioral); *location* (year, country, WHO region); *host population*: type (human, animal, plant) and setting (school/family, hospital, community, farm, etc.); *data*: data used for parameterization (epidemiological, clinical, behavioral, demographic, geospatial), data availability (public, on request, private); *methodology*: model class (compartmental or individual-based), inference method, and study type (explicative, predictive, interventions vs. forecasting); and *metadata* (authors, institutions, funding). Pathogen types were also later compared with the published WHO and center for disease control (CDC) lists of most urgent threats in AMR [1, 15].

### Time trend and impact analysis

A goal of our systematic review was to explore trends in the publication output for AMR modeling studies and their impact in the field, as AMR is emerging as a global health threat. Our review focused on the period 2006–2016; to explore publication trends in earlier years, we used a prior review by Temime et al. [11] which covered the period 1993–2006. Further, for comparison with a related area of infectious disease modeling, we compiled trends in publication of individual-based transmission models (defined as a model tracking the characteristics of an individual, including infection and transmission, over time), based on a recent systematic review [16]. In addition to the volume of AMR modeling publications, we assessed the impact of these publications in the field using the metric field-weighted citation impact (FWCI) [17]. The FWCI is the ratio between the number citations for a specific article and the average number of citations received by similar articles in the same field, type, and year of publication, thus making values comparable across these three variables. A FWCI greater than 1.0 indicates that publications have been cited more than would be expected; for example, a score of 1.2 means that an article has been cited 20% more than average. It should be noted that a FWCI score can vary over time and that data in our manuscript is based on a snapshot of the Scopus database taken on November 21, 2018.

### Intervention analysis

We used a seminal 2016 Review on Antimicrobial Resistance as a framework to classify interventions [18]. The report identified 10 intervention categories, of which only the first six were relevant to our study: (1) education or awareness campaigns, (2) improved hygiene and infection control, (3) reduction in use of antimicrobials, (4) improved surveillance of resistance, (5) improvement and development of rapid diagnostics, and (6) use of antimicrobial alternatives such as vaccines and alternatives. We also added a seventh category to consider antimicrobial regimen changes, as this is an area of high interest for public health (e.g., antimicrobial switching, cycling, introduction of new drug class).

Further, we identified whether interventions were modeled on a “micro” (institution level) or “macro” level (structural or policy interventions that might affect large populations, communities, countries, or regions). We also assessed whether the aim of the study was to prevent the development/acquisition of AMR (de novo resistance) or direct transmission of a resistant pathogen.

## Results

Details of the screening process can be found in the PRISMA diagram in Fig. 2. A total of 2466 articles were identified after removing duplicates. Two rounds of title and abstract screening removed a further 2143 records. A total of 323 articles were earmarked for full-text review. Upon reading these, we found that 50 articles did not meet the inclusion criteria specified above, which resulted in a final tally of 273 records included in our analyses. We describe the characteristics of all studies below and then focus on key findings for the five pathogens or diseases most commonly modeled: methicillin-resistant *Staphylococcus aureus* (MRSA), tuberculosis (TB), human immunodeficiency virus (HIV), influenza, and malaria.

### Trends in the number of published modeling studies

**Fig. 3:**
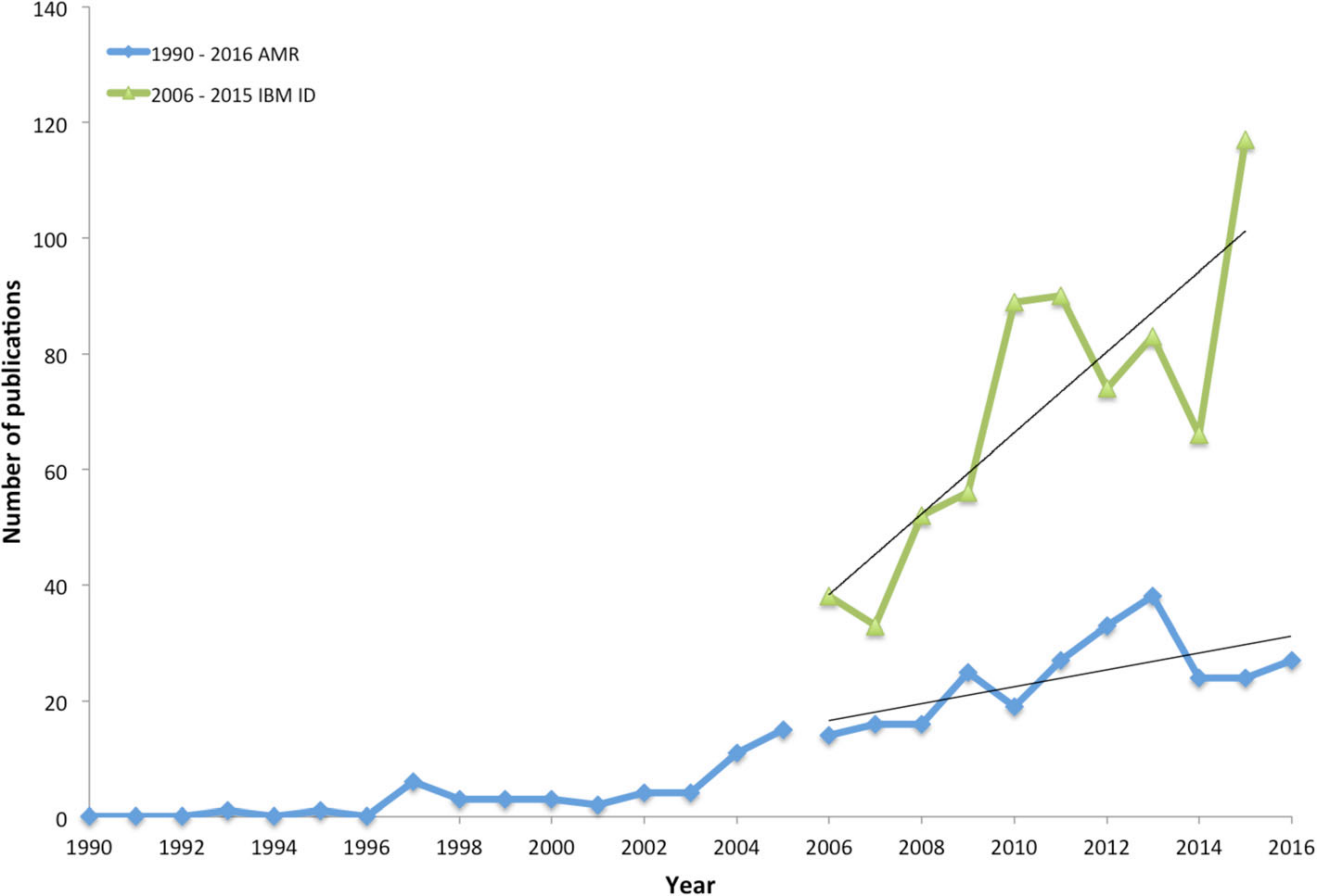
Yearly number of AMR modeling studies (1990–2016). This figure compares the yearly number of AMR modeling studies (based on data from Temime et al. (1990–2006) [11] as well our analysis (2006–2016), with the number of individual-based models used to analyze infectious disease (IBM ID) identified by Willem et al. between 2006 and 2015 [16]

We found an increasing trend (Fig. 3) in the annual number of AMR modeling studies between 2006 and 2016 (linear trend, slope = 1.5, *R*^2^ = 0.43), building off the steady increase shown by Temime et al. [11]. Since 2013, the pace of AMR modeling publications has leveled off at around 25 articles/year. In contrast, as described by Willem et al. [16], publications on individual-based models of infectious diseases have experienced a faster increase over the same time period (linear trend, slope = 7, *R*^2^ = 0.66), with on average three to four times more articles published on infectious disease related individual-based models than on AMR (Fig. 3). A histogram showing the number of AMR modeling articles published per year since 1990 can be found in Additional file 1: Fig. S1.

In addition to overall publication output, we assessed the influence of AMR modeling publications in the field using the FWCI score. The three publications with the highest FWCI during this period had a FWCI greater than 10 (two articles on TB [19, 20] and one on pandemic flu [21]). Excluding these three highly cited outliers, we found that the median FWCI for publications ranged between 0.47 and 2.65, with an overall median of 0.96, indicating that AMR modeling publications are being cited at a rate on par with other studies in their field (Additional file 1: Figure S2).

### Distribution of modeling studies by pathogen type

Approximately 65% of the AMR studies focused on bacterial diseases, 25% on viral diseases, 13% on parasitic diseases, and 2% on plant fungal pathogens. The top five pathogens most prominently studied were MRSA (25%), TB (16%), *Plasmodium falciparum* (8%), HIV (13%), and influenza (11%). For a detailed list of pathogens studied in each publication, see Additional file 1: Table S1. There was no significant time trend in the modeling of specific pathogens (Additional file 1: Figure S3).

### Host and population settings used in AMR modeling

Of the 273 publications considered in our review, 89% (*n* = 234) concerned human hosts, 7% (*n* = 18) focused on animal diseases, and 2% (*n* = 5) considered plant hosts. Only 2% (*n* = 6) addressed transmission between humans and animals in the same model. Animal transmission studies were mainly on animals of agricultural importance, although one explored transmission between humans and companion animals [22]. Only one study modeled the interaction of AMR pathogens between their hosts and the environment [23]. The majority of studies were either set exclusively in the community (*n* = 151, 55%) or in a healthcare facility (*n* = 74, 27%), with few (*n* = 11, 4%) exploring the link between these two (Table 1). Only eight studies (3%) modeled the transmission of AMR in long-term care facilities such as nursing homes, which are thought to be major reservoirs of AMR. The model populations were largely homogeneous and did not allow for variable mixing rates. A minority of the studies (*n* = 48, 18%) included heterogeneity in age, gender, sexual activity, and treatment status for pathogens such as TB, HIV, influenza, or malaria [24, 25]. Details can be found in Additional file 3: Table S4.

**Table 1:** Distribution of selected studies according to study characteristics.

	TOTAL	MRSA	TB	HIV	Influenza	Malaria
Host type	(*n* = 273)	(*n* = 65)	(*n* = 43)	(*n* = 34)	(*n* = 30)	(*n* = 22)
Human	233 (89%)	62 (95%)	43 (100%)	34 (100%)	28 (93%)	21 (95%)
Animal	18 (7%)	2 (3%)	–	–	2 (7%)	1 (5%)
Human-animal	7 (2%)	1 (2%)	–	–	–	–
Plant	5 (2%)	–	–	–	–	–
Population type						
Community (endemic)	154 (58%)	7 (11%)	40 (93%)	34 (100%)	25 (83%)	21 (95%)
Healthcare facility	71 (27%)	49 (75%)	1 (2%)	–	–	–
Community-healthcare facility	11 (4%)	5 (8%)	2 (5%)	–	1 (3.5%)	–
Agricultural-farming	20 (8%)	1 (1.5%)	–	–	1 (3.5%)	–
Other	8 (3%)	3 (4.5%)	–	–	3(10%)	1 (5%)
Model parameters						
Referenced data	191 (72%)	34 (52%)	38 (88%)	32 (94%)	29 (97%)	17 (77%)
Primary data	73 (28%)	31 (48%)	5 (12%)	2 (6%)	1 (3%)	5 (23%)
Model type						
Deterministic	175 (66%)	30 (46%)	36 (84%)	23 (70%)	18 (60%)	18 (82%)
Stochastic	57 (22%)	30 (46%)	6 (14%)	5 (12%)	3 (10%)	2 (9%)
Both (D and S)	25 (9%)	5 (8%)	1 (2%)	4 (12%)	8 (27%)	1 (5%)
Hybrid	7 (3%)	–	–	2 (6%)	1 (3%)	1 (5%)
Model class						
Compartmental	201 (76%)	34 (52%)	37 (86%)	27 (79%)	26 (87%)	19 (86%)
Individual-based	33 (12%)	19 (29%)	3 (7%)	4 (12%)	1 (3%)	1 (5%)
Both	7 (3%)	1 (2%)	–	2 (6%)	1 (3%)	1 (5%)
Other	23 (9%)	11 (17%)	3 (7%)	1 (3%)	2 (7%)	1 (5%)
Model features						
Multi-strain model	190 (72%)	23 (35%)	42 (98%)	32 (94%)	29 (97%)	17 (77%)
Co-infection of hosts	22 (8%)	5 (8%)	9 (21%)	5 (15%)	3 (10%)	3 (14%)
Fitness cost modeled	132 (50%)	15 (23%)	32 (74%)	20 (59%)	25 (83%)	13 (59%)
Acquired and transmitted resistance	89 (34%)	1 (2%)	26 (60%)	28 (82%)	20 (67%)	5 (23%)
Within-host model	17 (6%)	1 (2%)	3 (7%)	2 (6%)	1 (3%)	4 (18%)
Population stratification	48 (18%)	4 (6%)	11 (26)	18 (53%)	3 (10%)	4 (18%)
Model rigor						
Calibration	115 (43%)	33 (51%)	26 (60%)	16 (47%)	4 (13%)	3 (14%)
Validation	36 (14%)	10 (15%)	11 (26%)	5 (15%)	0 (0%)	1 (5%)
Sensitivity analyses	159 (60%)	36 (55%)	32 (74%)	25 (74%)	16 (53%)	14 (64%)
Economics						
Cost/benefit analysis	23 (9%)	8 (12%)	6 (14%)	6 (18%)	1 (3%)	3 (14%)

**Fig. 4:**
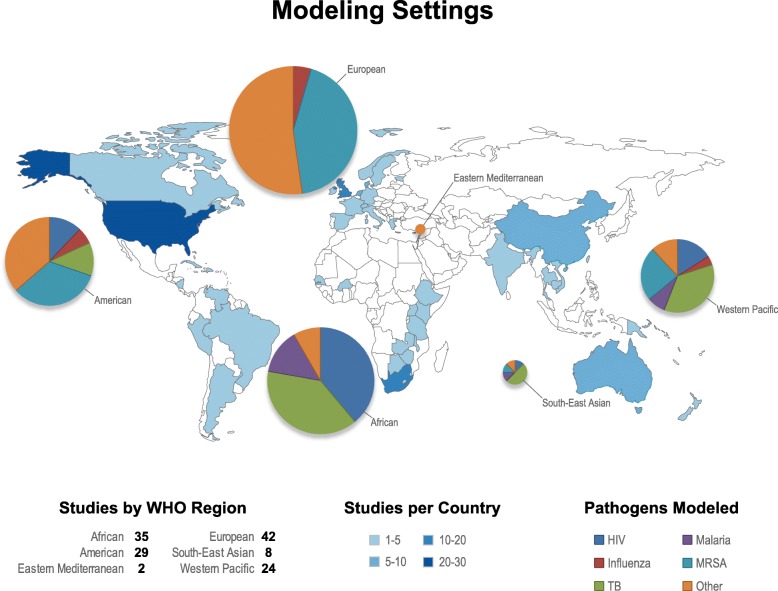
Geographic locations of models and pathogens modeled. A visual representation of 146 models that used parameters specific to geographic settings. One hundred seventeen models did not specify a particular geographic location. We also show the percentage of modeling studies by WHO region, categorized by the most highly represented pathogen types (HIV, human immunodeficiency virus; Influenza; Malaria; MRSA, methicillin-resistant *Staphylococcus aureus*; TB, tuberculosis). The size of the pie charts is proportional to the number of studies

A large fraction of studies (*n* = 121, 44%) did not focus on a particular geographic area. Those that did were approximately evenly split between four regions: Africa (*n* = 35, 13%), the Americas (*n* = 36, 13%), Europe (*n* = 43, 16%), and Western Pacific (*n* = 24, 9%) (Fig. 4). Few studies modeled AMR in either the Eastern Mediterranean (*n* = 2, 1%) or South East Asian (*n* = 8, 3%) regions. Most models that did specify a geographic location focused on only one country and did not model transmission between countries. Five studies modeled global transmission of the pathogen of interest [26–30]. There was an association between the pathogens modeled and country income status: 91% of studies (74/81) that specified locations and modeled HCAI were restricted to high-income countries (Table 2). On the other hand, the majority of TB and malaria modeling studies were set in low- and middle-income countries (LMIC) (Table 2). HIV was the only disease modeled across all regions (Table 2).

**Table 2:** Pathogens modeled by World Bank income level.

Infectious disease system	Total	High	Upper-middle	Low-middle	Low	ND
MRSA	65	38	2	1		24
TB	43	3	15	7		25
HIV	34	4	9	4	2	15
Influenza	30	5				25
*Plasmodium falciparum*	21		1	5	1	14
General bacteria	17	4				13
*Streptococcus pneumoniae*	12	6			1	5
*Enterococci* (VRE)	10	7				3
*Escherichia coli*	9	6	1			2
*Neisseria gonorrhoeae*	6	3				3
*Acinetobacter baumannii*	4	1	1			2
*Zymoseptoria tritici*	4	3				1
*Enterobacteriaceae* (ESBL-E)	3	2				1
*Klebsiella pneumoniae*	3	3				
*Teladorsagia circumcincta*	3	3				
*Campylobacter*	2	2				
General protozoan	2	1				1
Nematodes	2	1				1
*Pseudomonas aeruginosa*	2	2				
*Salmonella* spp.	2	1			1	
*Schistosoma mansoni*	2					2
*Blumeria graminis*	1	1				
*Enterobacteriaceae*, CRE	1		1			
*Leishmania donovani*	1			1		
NS	1					1
*Plasmodium chabaudii*	1					1
*Shigella* spp.	1	1				
*Trichostrongylus colubriformis*	1	1				
*Wucheria bancrofti*	1				1	

A representation of pathogens modeled by the World Bank income level classification: high, upper-middle, low-middle, low, or not described (ND).

### Modeling structure, dynamics, and model fitting

Of the 273 studies analyzed, most used deterministic models (*n* = 175, 66%). Other studies adopted stochastic models (*n* = 57, 22%), or hybrid deterministic models containing some elements of stochasticity (*n* = 7, 3%). A few studies compared the results of deterministic and stochastic methods (*n* = 25, 9.5%). Models were predominantly compartmental (*n* = 201, 76%) relative to individual-based models (*n* = 33, 12%). Several studies compared AMR outcomes using both model strategies (*n* = 7, 3%) (Table 1). A full breakdown of models by class is available in Additional file 1: Table S2.

Most studies considered more than one pathogen strain (*n* = 190, 72%), but the majority of the studies did not allow for co-infection of hosts, with a few exceptions (*n* = 22, 8%) (see Additional file 3: Table S4 for details). Half of the studies considered that the resistant strain carried a fitness cost (*n* = 132, 50%); however, fitness cost was often assumed, and few studies used primary data to infer this parameter (*n* = 21, 8%). With regard to the type of resistance studied, many models (*n* = 119, 45%) focused exclusively on transmitted resistance (secondary resistance) and significantly fewer models (*n* = 36, 14%) explored acquired or de novo resistance. Approximately a third of models (*n* = 89, *n* = 34%) accounted for both acquired and transmitted resistance, and some (*n* = 20, 8%) did not differentiate. Interestingly, a few studies integrated within- and between-host models (*n* = 17, 6%), allowing for joint exploration of emergence and transmission of AMR.

Model calibration against epidemiological or experimental data is an important feature of mathematical modeling. Some form of calibration (partial or full parameter calibration) was reported in just under half of the studies (*n* = 115, 43%). In addition to model calibration, sensitivity analysis testing the impact of varying parameter values on model outputs is critical to explore the robustness of conclusions. Out of 273 studies, 159 (60%) reported some level of parameter sensitivity or uncertainty analysis.

The accuracy of model results can also be assessed by out-of-sample validation techniques, in which model predictions are compared to independent observations that have not been used for model calibration. Only 36 studies (14%) reported out-of-sample model validation. From these, 31 used a statistical approach, while 5 simply conducted “face validity” tests by qualitative comparisons to empirical epidemiological datasets. There was no significant time trend in the type of models used, nor in the proportion of studies presenting a calibration or validation step (Additional file 1: Figure S4).

Finally, integration of economic frameworks in mathematical models to project economic costs can help to inform public health decision makers, by translating model results into more tangible cost-benefit analyses. Only 23 studies (*n* = 23, 9%) included financial components and proposed cost-benefit or savings analyses.

### Intervention analysis

Mathematical models can be particularly useful to assess the effectiveness of intervention strategies (Table 3). Studies modeling interventions were approximately evenly split between interventions targeting non-resistant pathogens (*n* = 99) and those aimed specifically at suppressing resistance (*n* =100). Several articles (*n* = 17) explored interventions that could be classified as being aimed at the suppression of both susceptible and resistant pathogens. Of those aimed at reducing resistance (*n* = 117), few (*n* = 20) focused on reducing the emergence or acquisition of resistance, while the majority (*n* = 82) focused on the transmission of resistant pathogens, and some (*n* = 15) considered both (Table 3). Perhaps unsurprisingly, the majority of models (*n* = 85) focused on micro-level interventions affecting institutions (such as hospital-level interventions), with fewer (*n* = 32) focusing on macro-level interventions such as national policy changes or vaccines (Table 3).

**Table 3:** Characteristics of AMR-specific interventions reviewed

Resistance target	
Acquired	20 (17%)
Transmitted	82 (70%)
Both	15 (13%)
Intervention scale	
Micro	85 (73%)
Macro	32 (27%)
Both	0
Recommended strategies to combat AMR	
1. Education or awareness campaigns	3 (3%)
2. Improved hygiene and Infection Control	59 (50%)
3. Reduction in use of antimicrobials	16 (14%)
4. Improved surveillance of resistance	32 (27%)
5. Improved and rapid diagnostics	10 (9%)
6. Vaccines and alternatives	11 (9%)
7. Changes to drug regimens	46 (39%)

We categorized the interventions from 117 studies that were specifically aimed at blocking AMR based on whether the interventions targeted acquired or transmitted resistance, the scale of interventions, and the type of intervention strategy modeled, motivated by the categories identified in a seminal report [18]. It should be noted that several models investigated the effects of more than one intervention; therefore, the sum of total strategies evaluated (*n* = 177) exceeds the total number of studies evaluated (*n* = 117).

We analyzed interventions based on the categories identified in a seminal report on AMR [18] (Table 3).The interventions studied were primarily improved hygiene or infection control measures (*n* = 59, 50%) such as hand hygiene, isolation, and decolonization. The impact of different drug regimens was often explored (*n* = 46, 39%) and included techniques such as mixing, switching, and cycling of drugs as well as changes to drug dosage and frequency. Surveillance of resistance (*n* = 32, 27%), rapid diagnostic techniques (*n* = 10, 9%), and a reduction in exposure to antimicrobials (*n* = 16, 14%) were also modeled. Relatively few studies included alternative treatment strategies or vaccines (*n* = 11, 9%). Only three studies modeled behavioral interventions (*n* = 3, 3%). Generally, many interventions modeled were organism specific, and further details can be found in Additional file 1: Table S3 and Additional file 3: Table S4.

### The five most common resistant pathogens modeled

We provide a short summary of the main findings of AMR modeling efforts for each of the top five diseases included in our review: MRSA, TB, HIV, influenza, and malaria.

### Methicillin-resistant *Staphylococcus aureus* (MRSA)

Almost all of the 58 MRSA transmission studies focused exclusively on humans, except for three that explored MRSA in animals or the associations between animals and humans [22, 31, 32] (Table 1). The studies were mainly set in healthcare facilities (*n* = 49, 75%), with a few modeling transmission between hospitals and other settings (*n* = 5, 8%). Only one model was set in low-middle-income country. Key findings of these studies include: (1) reaffirming the importance of hand hygiene compliance; (2) the prediction of coexistence of community-acquired and hospital-acquired MRSA [33–35], rather than the dominance of one over the other (although Webb *et al.* predict that community-acquired MRSA will dominate [36]); (3) the importance of effectively implementing appropriate screening, followed by isolation and/or decolonization; (4) the importance of hygiene and infectious disease control measures; and finally (5) two studies that proposed the intriguing concept of vaccines as a new weapon against MRSA [37, 38].

### Tuberculosis

We identified a total of 43 models studying the dynamics of TB resistance in humans, mainly in community settings (*n* = 40, 93%). The studies modeled general transmission dynamics of multidrug-resistant (MDR) or extensively drug-resistant (XDR) TB and considered multiple interventions, most commonly intermittent preventative therapy (IPT); directly observed treatment, short-course (DOTS); and surveillance and drug susceptibility testing (Additional file 1: Table S3). Major conclusions include the following: (1) the vast majority of MDR-TB incidence is due to transmitted resistance rather than de novo treatment-related acquisition [30, 39, 40]; (2) to combat resistance, drug susceptibility testing and TB surveillance should be emphasized [41–44]; (3) treatment and drug susceptibility testing should be expanded in community settings in Africa and the private sector in India [42, 43, 45–47]; (4) controlling HIV would help decrease the transmission rates of resistant -TB [48, 49]; (5) isolation or quarantine strategies would help prevent transmission and decrease the number of patients lost to follow-up [50, 51]; and (6) while community-wide intermittent preventative therapy may increase the incidence of drug resistance, the benefits in reducing primary TB infections outweigh the risks. However, such therapy should be coupled with appropriate diagnostic and treatment policies [48, 52–54].

### Human immunodeficiency virus

HIV studies represented 13% of our data (*n* = 34). Topics modeled included the dynamics of HIV resistance in the context of the introduction of new pharmaceutical interventions (e.g., antiretroviral therapy, pre-exposure prophylaxis, vaginal microbicides, or structural interventions such as changes in diagnostics or treatment policy (Additional file 1: Table S3)). Seven additional papers modeled HIV-TB co-infection. Several manuscripts reached similar conclusions, most notably the following: (1) while oral pre-exposure prophylaxis is expected to reduce new HIV infections, a rise in de novo resistance is projected if prophylaxis is administered to those unknowingly infected with HIV [55–62]; (2) similar findings apply to vaginal microbicides [63–65]; and (3) modeling stresses the likelihood of accumulation of resistance over time as a response to various therapies and the importance of regular viral load testing and early diagnosis [66–69]. Various changes in HIV treatment policy or diagnostics were also modeled [66, 68–75].

### Influenza

Influenza resistance modeling studies (*n* = 30) mostly focused on humans, with few exceptions (one transmission model in chickens and one between ferrets) [76, 77]. Interventions modeled included use of antivirals (matrix ion channel or neuraminidase inhibitors), vaccines, antibiotics for treatment of secondary infections, and non-pharmaceutical interventions (isolation and social distancing) (Additional file 1: Table S3). Three repeating themes emerged: (1) there is support for the use of prophylactic drugs despite the risk of developing resistance during pandemic situations, but conditions varied [21, 78–85]; (2) timing, dosage, and coverage levels of drugs are important when it comes to determining treatment effectiveness [82–91]; and (3) there is a need for monitoring the transmissibility and/or fitness of the resistant virus [28, 77, 78, 92–94].

### Malaria

A total of 22 studies described mathematical models for transmission of *Plasmodium* species in the context of AMR. All studies modeled *Plasmodium falciparum* in humans with the exception of one study of *Plasmodium chabaudi* in mice [95]. Geographically defined studies were restricted to Sub-Saharan Africa and the Thai-Cambodian region. Pharmaceutical interventions included the following drugs: artemisinin or artemisinin combination therapy (ACT), chloroquine, sulphadoxine, and pyrimethamine. Various non-pharmaceutical interventions were also modeled (Additional file 1: Table S3). Major conclusions include (1) the importance of using artemisinin as part of combination therapy regime (rather than monotherapy) [25, 96–99] and (2) intermittent preventive therapy should be used carefully in areas where resistance is not already established [24, 100].

## Discussion

Our systematic review of transmission modeling of AMR over a decade highlights a continuous increase in publications during 1996–2012, a peak in 2013 (*n* = 38), and a plateau in the following 3 years (average annual publications = 25). Modeling of AMR overall experiences a slower progression than a related field such as individual-based infectious disease models. Five infectious diseases have dominated mathematical models of AMR during 2006–2016: MRSA, TB, HIV, influenza, and malaria. The majority of AMR articles focused exclusively on humans, either in community or healthcare settings, rather than modeled interactions between hosts or multiple settings. Over the study period, a majority of models remained data-free and few were validated against independent datasets. Many models assumed a fitness cost for resistant organisms; however, this was often not derived from primary experimental or epidemiological data. Few models integrated within-host dynamics or economic factors into their transmission framework. Most of the interventions aimed at combating AMR were primarily focused on transmitted rather than acquired resistance and were implemented on a micro-level scale. The interventions considered the impact of different drug regimens, hygiene and infection control measures, or screening and diagnostics, while less than 5% addressed alternative therapeutic strategies or behavioral changes.

The predominance of five pathogens in AMR transmission modeling is likely driven by a long history of disease modeling for at least four of these pathogens (TB, HIV, influenza, and malaria) and an early recognition of MRSA as an important drug-resistant pathogen, combined with availability of epidemiological and surveillance data. Historically, these diseases have taken a large toll on global morbidity and mortality rates; however, it has been predicted that the consequences of AMR in other pathogens may rapidly outpace them by 2050 [18]. More research should be undertaken before resistance in other disease systems becomes a major crisis.

The observed skew towards these 5 diseases was in stark contrast in comparison to the WHO’s priority list of 12 antibiotic resistant bacteria [1] and the CDC’s list of 18 drug-resistant threats in the USA [15]. Only a handful of studies modeled the diseases categorized as the most urgent by the WHO and CDC: *Neisseria gonorrhoeae* (*n* = 6), *Acinetobacter baumannii* (*n* = 4), ESBL-producing enterobactriaceae (*n* = 3), *Pseudomonas aeruginosa* (*n* = 2), carbapenem-resistant enterobactriaceae (*n* = 1), and *Clostridium difficile* (*n* = 0) (Table 4). The lack of *Clostridium difficile* resistance studies is puzzling as several mathematical models exist for sensitive strains of this pathogen (e.g., [101]). In contrast, the top two bacteria represented in our AMR review, MRSA and TB, were the focus of 65 and 43 studies respectively. And while modeling of an intermediate-level threat like vancomycin-resistant enterococci is gaining momentum (*n* = 10), much remains to be done to understand transmission in the community and environmental settings.

**Table 4:** The number of modeling studies compared to the WHO and CDC lists of important AMR threats.

Pathogen	WHO category	CDC category	AMR models	References
*Enterobacteriaceae*, carbapenem-resistant	C1	C1	1	[156]
*Enterobacteriaceae*, ESBL-producing	C1	C2	3	[141, 157, 158]
*Acinetobacter baumannii*, carbapenem- or multidrug-resistant	C1	C2	4	[158–161]
*Pseudomonas aeruginosa*, carbapenem-or multidrug-resistant	C1	C2	2	[139, 158]
*Neisseria gonorrhoeae*, cephalosporin- or fluoroquinolone-resistant	C2	C1	6	[162–167]
*Staphylococcus aureus*, methicillin-resistant	C2	C2	65	[22, 31–38, 151, 158, 168–221]
*Enterococcus faecium*, vancomycin-resistant	C2	C2	10	[158, 202, 222–229]
*Campylobacter* spp., fluoroquinolone-resistant	C2	C2	2	[229, 230]
*Salmonellae*, (Typhi and non-typhoidal), fluoroquinolone-resistant	C2	C2	2	[231, 232]
*Staphylococcus aureus*, vancomycin-intermediate and resistant	C2	C3	–	–
*Helicobacter pylori*	C2	–	–	–
*Haemophilus influenzae*, ampicillin-resistant	C3	–	–	–
*Streptococcus pneumoniae*, penicillin-non-susceptible	C3	C2	12	[37, 233–240]
*Shigella* spp., fluoroquinolone-resistant	C3	C2	1	[241]
*Clostridium difficile*	–	C1	–	–
*Mycobacterium tuberculosis*, MDR, XDR	–	C2	43	[19, 20, 30, 39–54, 242–265]
Fluconazole-resistant *Candida*	–	C2	–	–
*Streptococcus agalactiae*, clindamycin-resistant group B MDR, XDR	–	C3	–	–
*Streptococcus pyogenes*, erythromycin-resistant group A MDR, XDR	–	C3	–	–

The pathogens that pose the greatest threat to human health according to the WHO and the top drug-resistant threats in the USA according to the CDC. Category 1 (C1) threats are described as “Critical” (WHO) or “Urgent” (CDC); category 2 (C2) as “High” (WHO) or “Serious” (CDC); and category 3 (C3) as “Medium” (WHO) or “Concerning” (CDC).

Other serious threats based on WHO or CDC criteria that are rarely modeled include *Campylobacter* (*n* = 2), *Salmonellae* spp*.* (*n* = 2), *Neisseria gonorrhoeae*, and *Shigella* spp. (*n* = 1). Importantly, we were unable to find any published AMR models for the following serious threats: *Helicobacter pylori*, *Haemophilus influenzae*, fluconazole-resistant *Candida*, clindamycin-resistant group B strep, and erythromycin-resistant group A strep. While mathematical transmission models do exist for wild-type *H. pylori* [102], *H. influenzae* [103], and *Candida parapsilosis* [104], we are not aware of any models for resistant strains, which may have different transmission parameters than susceptible strains.

Most models did not consider pathogen heterogeneity, such as multiple viral or bacterial strains, parasite species, or multiple resistance mechanisms (e.g., membrane permeability, enzymatic degradation, mutation of antimicrobial targets), which might affect transmission potential. As a case in point, most malaria modeling has dealt with the *Plasmodium falciparum* species in Africa or East Asia. This is presumably based on the long-held assumption that the majority of malaria burden is caused by *P. falciparum* rather than other plasmodium species. However, there is growing evidence that *Plasmodium vivax*, which is endemic in South and South-East Asia as well as Central and South America, is associated with a significant burden of morbidity and associated mortality [105, 106]. *P. vivax* is already largely resistant to chloroquine [107], though resistance to artemisinin has not yet been reported. A similar issue exists in regard to mathematical modeling studies of HIV, where no distinction was made between HIV-1 and HIV-2, which are known to have markedly different resistance profiles to the various antiretroviral drugs used [108, 109]. This is likely because HIV-2 has historically infected a much smaller, but significant, proportion of the population. It was estimated in 2006 that one to two million people [110] in several West African countries were infected with HIV-2, though we could not find more recent estimates.

While there has been increasing effort to design models with explicit interactions between community and hospital populations, few include long-term care facilities, which often lack effective antimicrobial stewardship programs [111–113]. Most worrisome perhaps, almost all models were set in humans and there were few attempts to tackle the hypothesized connection between veterinary/agricultural use of antibiotics and AMR. No studies modeled AMR transmission in aquaculture, despite the growing body of evidence that AMR resistance could enter the food chain through these means [114, 115]. Similarly, there were few ecological studies on the transmission of AMR from the environment (water, soil, etc.) to potential hosts, despite the increasing evidence for a link between antimicrobial contamination of the environment, and the development and transfer of resistance to human pathogens [116–118]. This is particularly concerning given the large quantity of antibiotics used in agricultural facilities, the lack of regulation on their waste disposal and the inability of many sanitation systems to filter out antimicrobials and AMR elements. Another environmental factor that was not modeled was the effect of climate change on the rates of AMR. Recent research has shown that increasing temperatures are associated with increased levels of resistance [119, 120], but there is no projection of AMR patterns under climate change scenarios.

We found that the vast majority of HCAI and influenza models were set in high-income countries, although this is an increasingly recognized threat in LMIC [1]. The lack of studies in developing countries is particularly concerning because of unregulated or poorly regulated antimicrobial manufacturing and usage [121, 122]. This is likely due to lack of appropriate diagnostics and surveillance in low-resource settings [1, 122].

A major reason for the lack of modeling studies on particular pathogens or certain settings is likely to be a deficiency in available data needed for model calibration and design. There is a need for more precise data on antibiotic consumption rates in both humans and animals [18], which is often not made publicly available [123–125]. In addition, improved surveillance of AMR incidence is required in humans, animals, and the environment (soil and water) [126]. There have been several examples of zoonotic transmission of AMR in both domestic [127, 128] and wild animals [129, 130] as well as evidence of transmission of genetic determinants of AMR into the environment [3, 116], which in turn may facilitate further dissemination of resistance.

In terms of AMR-specific model dynamics, half of the reviewed studies factored in a fitness cost for the resistant strain; however, this was often assumed and rarely estimated from primary data. Additionally, many models did not distinguish between acquired (de novo) or transmitted resistance. This is important for accurately defining model parameters such as reversion [131] or transmission rates [78, 132], which ultimately affect model outcomes. Most studies modeled homogeneous infections with a single pathogen strain and therefore did not investigate host co-infection and strain competition. Host populations were also largely assumed to be mixing homogeneously with no stratification by age, susceptibility, or contact patterns. Integration of within- and between-host models was also rare; multi-scale modeling is an important frontier for AMR and more broadly for the field of infectious disease modeling [133].

Previous reviews predicted that technological advances in computational tools could allow for more complex models and calibration to larger datasets [9, 13]. Consistent with this prediction, a sharp increase was reported in the field of individual-based models of infectious diseases, but this increase has not percolated to the field of AMR [16]. The majority of AMR transmission models reviewed here remain theoretical, with little attempt to compare model predictions to epidemiological data, and calibration with independent data is scarce. It should also be noted that improvements could also be made in terms of documenting modeling methods. Only 47% of the studies assessed cited the modeling software or computational tools used and few described modeling techniques in a way that might be able to be reproduced by researchers who are not already experienced modelers. Even fewer manuscripts provided the computational code used: two manuscripts provided a link (both were expired at the time of this writing), and three were willing to share the code upon request. Some attempts have been made to standardize the terminology, methodology, and reporting structure for infectious disease transmission models [134–136], but better documentation of modeling methods is needed for reproducibility. Furthermore, it would also be useful to make the underlying AMR epidemiological datasets publicly available to aid reproducibility.

With regard to interventions aimed at combating AMR, many models incorporated elements of improved hygiene or infection control in order to combat the spread of AMR. No model focused on “macro” scale interventions such as improved access to water and sanitation facilities that can curb the transmission and development of resistance. Improved water, sanitation, and hygiene can lead to a decrease in respiratory and diarrheal disease, both of which are often unnecessarily treated with antibiotics although the causative agents may be viral [137, 138]. Numerous interventions examined improved surveillance or diagnostic methods, particularly for HIV and TB, but were lacking for many bacterial diseases outside of healthcare settings. Many diagnostic methods for antimicrobial resistance are culture based, and confirmation of resistance, let alone specific genotyping, may take several days. There is an urgent need for rapid molecular diagnostics in order to improve antimicrobial stewardship; more modeling work in this area could highlight the transmission and cost-effectiveness benefits of such technologies.

Surprisingly, few studies modeled reduction in the use of antimicrobials as an intervention, particularly when supplied to food animals either as a growth supplement or prophylaxis. Several models studied the effects of reducing antimicrobial exposure levels in healthcare settings [139–142], but there were fewer for animals [143–145]. No models for AMR or AMR-related interventions in aquaculture settings exist.

Many infectious disease models increasingly incorporate features of human behavior [123–125, 146]; however, this is not common in the field of AMR modeling outside of healthcare facilities. In addition, most models did not consider how social, cultural, or behavioral differences might affect resistance development or transmission. Those that did were mainly focused on sexually transmitted infections such as HIV or *N*. *gonorrhoeae.* Similarly, few models included vaccination despite increasing appreciation for the role they could play in reducing antimicrobial consumption [147, 148]. Vaccines can also have indirect effects on antimicrobial consumption [147, 148] by reducing the number of pharmaceuticals erroneously prescribed for viral infections. Several vaccine candidates are under development for *C. difficile*, *S. aureus*, group B *Streptococcus*, *E. coli*, and respiratory syncytial virus [149]; mathematical models could be used to evaluate their potential effects at a population level and inform cost-effectiveness analyses.

The increasing availability of multiple epidemiological and pathogen genetic data streams offers exciting new possibilities to improve and expand modeling capabilities. Enhanced access to, and integration of, digital disease surveillance data [150] into epidemiological analyses could help further strengthen model validation. Pathogen genomic sequences (together with relevant metadata such as date, location) can also inform many aspects of transmission dynamics. And although some have started integrating genomic data [151] into modeling studies, this is the exception rather than the norm in the field of AMR. An integrative approach will be required to synthesize large amounts of data together, which will ideally help to develop more realistic AMR models tailored to specific populations. It is noteworthy that few publications addressed the spatial diffusion of AMR; a lack of spatially resolved AMR datasets may explain this gap.

This review has some limitations. We have only searched four databases most relevant to biomedical sciences. Furthermore, in an effort to keep the amount of search results to a manageable number, we use certain keywords specific to population dynamic studies of AMR organisms. Therefore, we may have inadvertently excluded some publications (without these keywords) relevant to this review. However, we are confident that this review provides an accurate overview of overall trends in the field.

## Conclusions

The field of AMR modeling is growing but is limited by both the quantity and quality of available data. Success stories include accurate predictions of the emergence of resistance in malaria [152], MDR-TB [153], and influenza [154], and modeling is also frequently used to inform AMR stewardship programs in healthcare facilities [155]. Our review suggests a need for more applied, data-driven models, better tuned to and diversified to reflect the public health concerns highlighted by the WHO and the CDC. Although the overall increase in AMR transmission modeling in the last decade is encouraging, the recent plateau in published work and scarcity of studies on high-concern pathogens should be addressed. Most importantly perhaps, more forward-thinking models should be developed to predict the emergence of resistance in pathogens where the issue is not yet rampant and evaluate how policy and behavioral changes can curb drug pressure and mitigate AMR. Research programs in support of AMR modeling, increased data collection efforts, and stronger links between modelers and public health experts are warranted to stimulate this field.

Box 1: Challenges to mathematical modeling of AMR Data gaps:

- Lack of sufficient data on antimicrobial use in humans and animals, antimicrobial environmental contamination, and resistance rates in unmonitored industries and low-income countries.
- Lack of standardization in data definitions or collection methods.

Complexity of model dynamics:

- Lack of understanding of disease ecological dynamics or model too complex.
- Pathogen heterogeneity: resistance governed by multiple genetic and epigenetic factors, so that a diversity of strains can exhibit the same resistance phenotype (single nucleotide polymorphisms, acquisition or deletion of genes or plasmids, up- or downregulation of genes).
- Dynamic fitness landscapes: resistance carries fitness costs that are poorly understood and can decrease transmission potential, while compensatory mutations can restore transmission.
- Co-infection dynamics between sensitive and resistant strains: strain coexistence, competition, conversion, or replacement are possible depending on the disease studied.

Model assessment:

- Validation cannot take place without proper surveillance data.
- Inability to accurately evaluate AMR interventions in the field for ethical, practical, or political reasons.
- Inability to validate model parameters in a changing environment (changes in transmission rates, fitness costs, and growth potential under antibiotic treatment, as resistance evolves).

## Additional files

Additional file 1: **Figure S1.** Trend in the number of AMR models per year. **Figure S2.** (a) Field-weighted citation impact (FWCI) of AMR publications by year (excludes 3 publications with FWCI greater than 10) and (b) FWCI by pathogen category: viral (V), bacterial (B), parasitic (P), and fungal (F). **Figure S3.** Pathogen trends over time. The proportion of articles on selected infectious diseases relative to the total publications per year published between 2006 and 2016 (articles/year): healthcare-acquired infections (HCAI), methicillin-resistant staphylococcus aureus (MRSA), tuberculosis (TB), human immunodeficiency virus (HIV), influenza and plasmodium falciparum (malaria). **Figure S4.** Model characteristic trends over time: The proportion of articles with a particular model approach (deterministic or stochastic, compartmental or individual-based models) or rigor (calibrated or validated) by the total publications per year published between 2006 and 2016.** Table S1.** References for publications divided by diseases they model.** Table S2.** References for publications divided by model class. **Table S3.** References for publications divided by disease specific interventions. (DOCX 2019 kb)

Additional file 2: Preferred Reporting Items for Systematic Reviews and Meta-Analyses (PRISMA) 2009 checklist. (DOC 62 kb)

Additional file 3: **Table S4.** Complete table of data extracted from each publication included in this review. (XLSX 203 kb)

## Abbreviations

ACT: Artemisinin combination therapy
AMR: Antimicrobial resistance
CDC: Center for Disease Control and Prevention
ESBL: Extended spectrum beta-lactamases
FWCI: Field-weighted citation impact
HCAI: Healthcare-acquired infections
HIV: Human immunodeficiency virus
IBM: Individual-based model
LMIC: Low- and middle-income countries
MDR or XDR TB: Multidrug- or extremely drug-resistant tuberculosis
MRSA: Methicillin-resistant *Staphylococcus aureus*
ND: Not described
NS: Non-specific
PRISMA: Preferred Reporting Items for Systematic Reviews and Meta-analyses
TB: Tuberculosis
WHO: World Health Organization
