# Supplementary material for: Population-level mathematical modeling of antimicrobial resistance: a systematic review
Source: BMC Med. 2019 Apr 24;17:81. doi: 10.1186/s12916-019-1314-9 (PMC6480522; doi:10.1186/s12916-019-1314-9)
Supplement: Supplementary file 1 — Figure S1. Trend in the number of AMR models per year. Figure S2. (a) Field-weighted citation impact (FWCI) of AMR publications by year (excludes 3 publications with FWCI greater than 10) and (b) FWCI by pathogen category: viral (V), bacterial (B), parasitic (P), and fungal (F). Figure S3. Pathogen trends over time. The proportion of articles on selected infectious diseases relative to the total publications per year published between 2006 and 2016 (articles/year): healthcare-acquired infections (HCAI), methicillin-resistant staphylococcus aureus (MRSA), tuberculosis (TB), human immunodeficiency virus (HIV), influenza and plasmodium falciparum (malaria). Figure S4. Model characteristic trends over time: The proportion of articles with a particular model approach (deterministic or stochastic, compartmental or individual-based models) or rigor (calibrated or validated) by the total publications per year published between 2006 and 2016. Table S1. References for publications divided by diseases they model. Table S2. References for publications divided by model class. Table S3. References for publications divided by disease specific interventions. (DOCX 2019 kb) [file 12916_2019_1314_MOESM1_ESM.docx]

**SUPPLEMENTARY MATERIAL
Literature search terms:** The following query was used to search relevant databases: *("Antimicrobial resistance" OR “antimicrobial resistant” OR "Drug resistance" OR “drug resistant” OR Multidrug-resistant OR “multi-drug resistant” OR “multidrug resistance” OR “multi-drug resistance” OR “extensive drug resistance” OR "Extensive drug-resistant" OR "Carbapenem-resistant" OR “carbapenem resistant” OR “carbapenem resistance” OR “erythromycin resistant” OR “methicillin resistant” OR “fluconazole resistant” OR “vancomycin resistant” OR ((drug OR anti-infective OR antimicrobial OR antibiotic OR antibacterial OR microbicides OR antipseudomonal OR antifungal OR antiviral OR Anthelmintics OR antiparasitic OR vermifuges OR antiprotozoal OR antimalarial) AND (resistance OR resistant))) AND (infection OR disease OR parasite OR malaria OR bacteria OR virus OR "Clostridium difficile" OR “Clostridium Difficile” OR “c. difficile” OR “c. diff” OR “pseudomembranous colitis” OR Enterobacteria OR Enterobacteriaceae OR “carbapenem resistant Enterobacteriaceae” OR Salmonella OR “Salmonella enterica” OR “S. enterica” OR “Salmonella typhi” OR “S. typhi*” OR “Salmonella typhimurium” OR paratyphi OR “Non-typhoidal Salmonella” OR “Salmonella typhosa” OR “Escherichia coli” OR “E. coli” OR “Klebsiella pneumoniae” OR “K. pneumoniae” OR Klebsiella OR “Carbapenem-resistant Klebsiella pneumoniae” OR Shigella OR “S. dysenteriae” OR “S. flexneri” OR “S. boydii” OR “S. sonnei” OR Serratia OR “S. marcescens” OR citrobacter OR proteus OR “neisseria gonorrhoeae” OR “N. gonorrhoeae” OR acinetobacter OR “multidrug resistant acinetobacter” OR “multi drug resistant acinetobacter” OR “acinetobacter baumannii” OR “A. baumannii” OR “MDR acinetobacter baumannii” OR “MDR Ab” OR campylobacter OR “Campylobacter jejuni” OR “C. jejuni” OR “Campylobacter coli” OR “C. coli” OR candida OR candidiasis OR “C. albicans” OR “C. glabrata” OR “C. tropicalis” OR “C. parapsilosis” OR “Extended-spectrum beta-lactamase” OR “ESBL-producing Enterobacteriaceae” OR “Extended-spectrum Enterobacteriaceae” OR enterococ* OR “vancomycin-resistant enterococci” OR “vancomycin-resistant enterococcus” OR “E. faecalis” OR “E. faecium” OR “E. raffinosus” OR “E. mundtii” OR “pseudomonas aeruginosa” OR “P. aeruginosa” OR “staphylococcus aureus” OR “Methicillin-resistant Staphylococcus aureus” OR MRSA OR “S. aureus” OR “Vancomycin-resistant Staphylococcus aureus” OR VRSA OR “streptococcus pneumoniae” OR “s. pneumoniae” OR pneumococc* OR streptococcus OR “Erythromycin-resistant group A streptococcus” OR “Group A Streptococcus” OR “Clindamycin-resistant group B streptococcus” OR “Group B Streptococcus” OR Tuberculosis OR TB OR “Mycbacterium tuberculosis” OR “M. tuberculos” OR malaria OR plasmodium OR HIV) AND (“Mathematical model” OR “computational model” OR “Stochastic model” OR “statistical model” OR “deterministic model” OR “dynamical model”) AND (mathematic* OR stochast* OR deterministic OR discrete OR compartment* OR computation* OR statistic* OR transmission OR reproductive OR R0 OR reservoir OR estimat* OR epidemic OR epidemic* OR dynamic* OR simulat* OR “epidemic model” OR SIR OR SEIR OR SVIR OR “reproductive number” OR “epidemic modeling”).* This query was modified as necessary for searches in other databases.

**Figure S1**

**
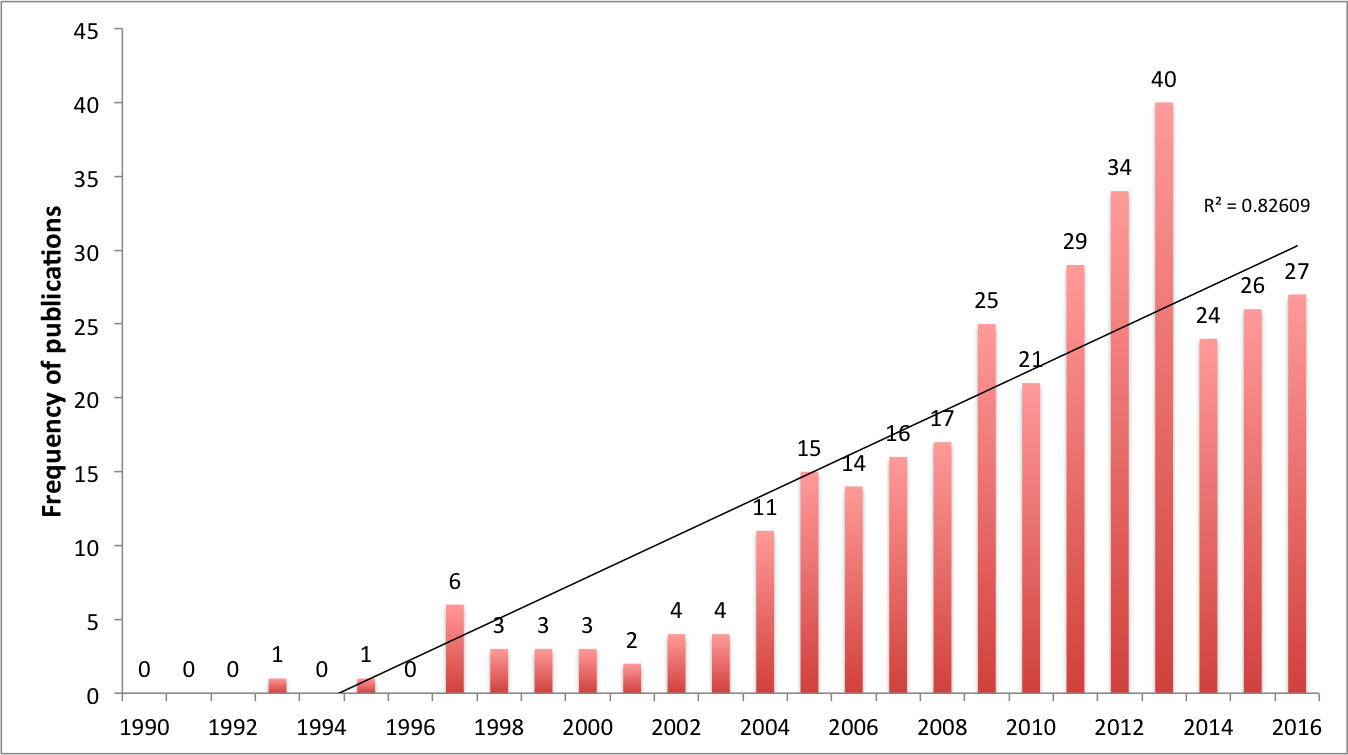
Figure S2
(a)**

**
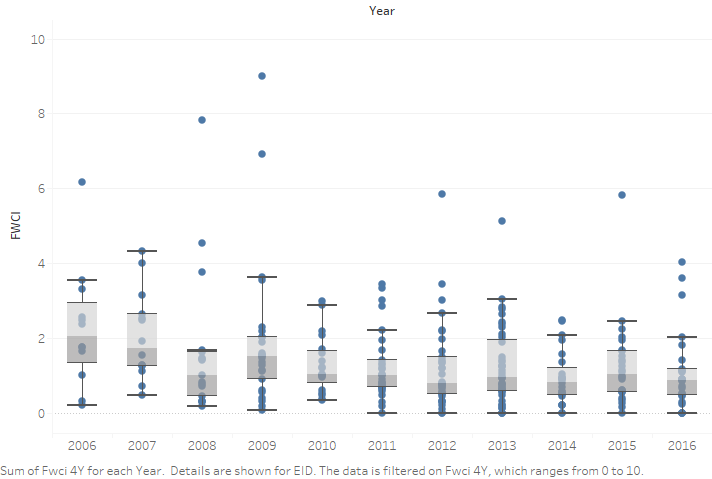
**

**(b)**

**
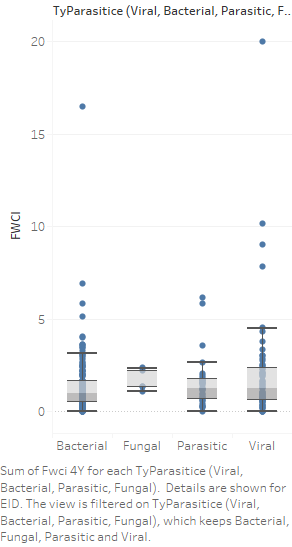
Figure S3**

**
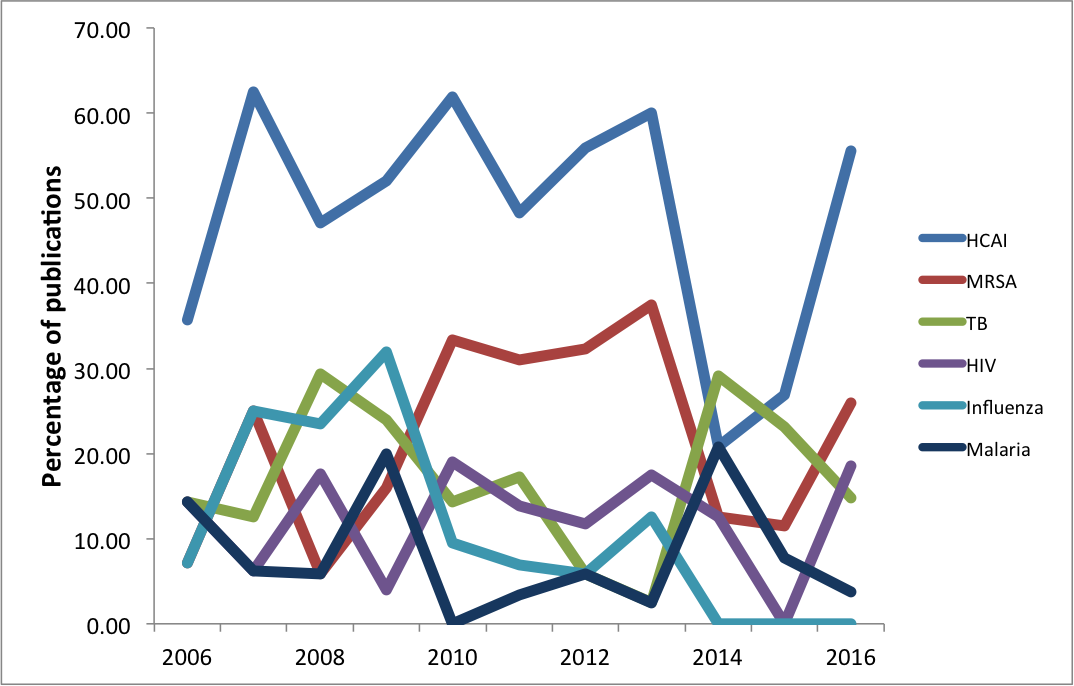
**

**Figure S4**

**
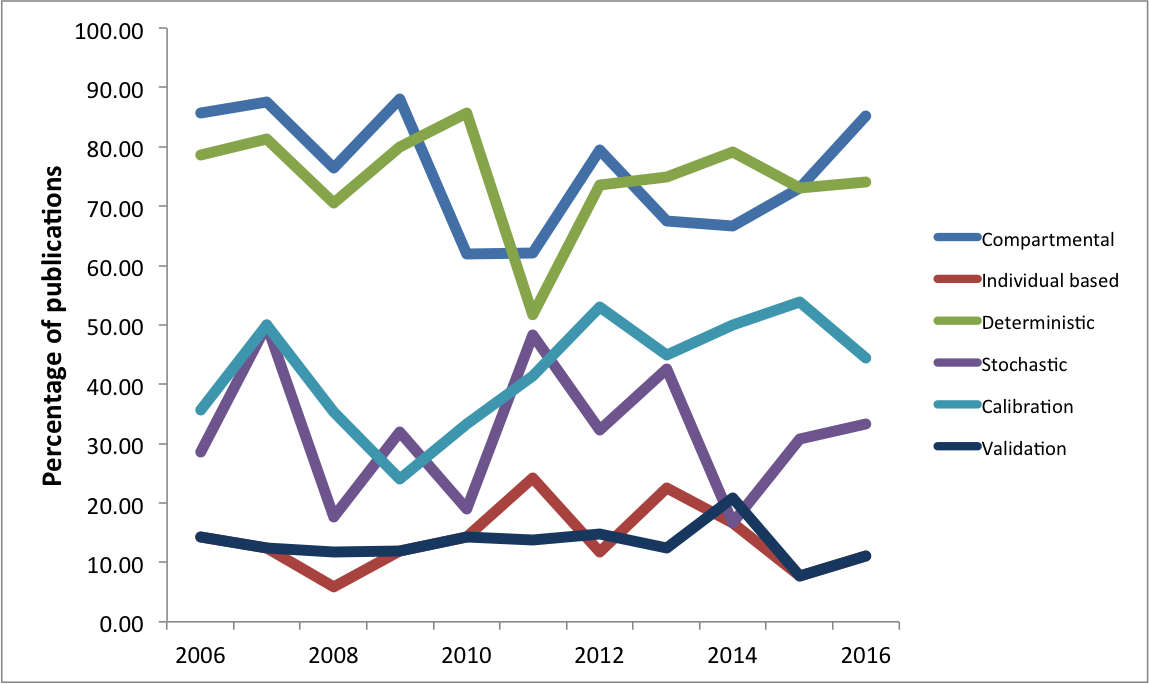
 Table S1: References for publications divided by diseases they model.**

| **Pathogen group** | **Pathogen Type** | **Reference** |
| --- | --- | --- |
| Bacteria | Acinetobacter baumannii | [1-4] |
|  | Campylobacter | [5, 6] |
|  | Enterobacteriaceae (CRE) | [7] |
|  | Enterococci (VRE) | [4, 6, 8-15] |
|  | Escherichia coli | [6, 16-23] |
|  | ESBL-E | [4, 24, 25] |
|  | General bacteria | [26-42] |
|  | Klebsiella pneumonia | [4, 16, 43] |
|  | MRSA | [4, 32, 44-106] |
|  | Mycobacterium tuberculosis | [107-149] |
|  | Neisseria gonorrhoeae | [150-155] |
|  | Pseudomonas aerginosa | [4, 156] |
|  | Salmonella enterica | [157, 158] |
|  | Shigella | [159] |
|  | Streptococcus pneumoniae | [106, 160-168] |
|  | General Parasites | [169, 170] |
|  | Leishmania donovani | [171] |
| Parasite | Nematodes | [172, 173] |
|  | Plasmodium chabaudi | [174] |
|  | Plasmodium falciparum | [106, 160-167, 175, 176] |
|  | Schistosoma mansoni | [177, 178] |
|  | Teladorsagia circumcincta | [179] [180] [181] |
|  | Trichostrongylus colubriformis | [179] |
|  | Wucheria bancrofti | [182] |
| Virus | HIV | [183-216] |
|  | Influenza | [217-245] |
| Fungus | Blumeria graminis | [246] |
|  | Zymoseptoria tritici | [247-250] |
| Non specific | Non specific | [251] |

**Table S2: References for publications divided by model class.**

| **MODEL CLASS** | **Number of studies** | **References** |
| --- | --- | --- |
| Compartmental model | 203 | [1, 3, 5, 7, 8, 10-12, 14-17, 19-21, 23-47, 51, 52, 54, 56, 58, 59, 61, 67-69, 72-74, 77, 79, 82, 84, 85, 87, 91-95, 98-102, 105-112, 114-122, 125, 127-129, 131-134, 137-144, 146-151, 153-156, 158, 160, 162-165, 167, 168, 170, 171, 173-178, 182-195, 197-199, 201, 202, 204-208, 210-214, 216-222, 224-231, 233, 234, 237-239, 241-270] |
| Individual-based model | 43 | [13, 21, 27, 50, 53, 55, 57, 60, 63-66, 70, 71, 75-77, 80-82, 88, 96, 103, 113, 126, 135, 152, 161, 172, 179-181, 196, 201, 208, 209, 215, 232, 252, 271] |
| Statistical model | 17 | [4, 9, 48, 49, 62, 83, 86, 97, 104, 136, 157, 159, 166, 169, 200, 223, 272] |
| Probabilistic | 4 | [90, 124, 130, 273] |
| Geospatial statistics | 3 | [18, 78, 123] |
| Microsimulation model | 3 | [89, 145, 203] |
| Network model | 3 | [2, 22, 240] |
| Individual mixture transmission model | 1 | [236] |
| Diffusion model | 1 | [6] |
| Game-theoretic model | 1 | [235] |

**Table S3: References for publications divided by disease specific interventions.**

| **INTERVENTION** | | **VIRAL** | | **BACTERIAL** | | | | **PARASITIC** | | | **FUNGAL** | **UNKNOWN** |
| --- | --- | --- | --- | --- | --- | --- | --- | --- | --- | --- | --- | --- |
|  |  | HIV | Influenza | TB | MRSA | HCAI | Non-HCAI | Malaria | Helminthes | Other | Ascomycota |  |
| **Non-pharmaceutical** | Cleaning |  |  |  | [74, 84, 91, 92, 105] | [2, 3, 36] |  |  |  |  |  |  |
|  | Hand hygiene |  |  |  | [44, 45, 50, 51, 53, 54, 59, 67, 74, 91, 101, 105] | [2, 3, 7, 8, 11, 23, 25, 33, 36, 43] |  |  |  |  |  |  |
|  | Contact / barrier precautions |  |  | [109] | [59, 85, 90, 92, 105] | [1, 7, 11, 26, 33, 36, 81, 89] |  |  |  |  |  |  |
|  | Decolonization |  |  |  | [45, 51, 53, 54, 58, 60, 67, 70, 75, 85, 86, 90-94, 96, 104, 105] | [1] |  |  |  |  |  |  |
|  | Ventilation |  |  | [109] |  |  |  |  |  |  |  |  |
|  | Length of stay /treatment |  |  | [109] | [101, 105] | [3, 8, 12] |  |  |  |  |  |  |
|  | Improved HIV retention | [209] |  |  |  |  |  |  |  |  |  |  |
|  | Administrative |  |  | [143, 148] | [45, 53, 66, 67, 95, 105] | [2, 8, 25, 36, 43] |  |  |  |  |  |  |
|  | Isolation; |  | [237] | [109, 140, 264] | [45, 46, 52, 53, 57, 69-71, 74, 76, 86, 91-93, 95, 96, 101, 104] | [3, 8, 9, 24, 34, 43] |  |  |  |  |  |  |
|  | Quarantine |  |  | [109, 111, 125] |  |  |  |  |  |  |  |  |
|  | Insecticide |  |  |  |  |  |  | [252, 259, 262, 263, 265, 266, 268] |  |  |  |  |
|  | Other NPI | [199] | [244] | [117] |  | [12, 14, 15, 20] |  | [263] |  |  |  |  |
| **Pharmaceutical** | Drug availability | [186, 210, 211] |  | [111, 117, 143, 148] | [72] | [12, 18, 23, 25, 33, 40, 41, 43, 156] |  |  | [173] |  |  |  |
|  | Drug therapy | [183, 187, 189, 191, 192, 199, 200, 202, 203, 206, 207, 209, 215] | [218-221, 223-229, 234, 235, 237, 238, 241, 245] | [109, 116, 126, 127, 130, 134, 139, 141] | [56, 68, 73, 79, 88] | [14, 15, 39] | [5, 151, 152, 154, 155, 160, 166, 168] | [252, 255-259, 262, 263, 266, 269, 272] | [177, 178, 182] |  | [246-250] |  |
|  | Prophylaxis | [193, 194, 197, 202, 204, 205, 208, 212, 213] | [217, 219-221, 223, 229, 231, 232, 235, 239] | [126] |  | [26] |  |  |  |  |  | [251] |
|  | Drug regimen strategies |  | [233, 240, 242-244] | [108, 124, 138, 143, 148] | [77, 103, 260] | [12, 27, 29-32, 34, 35, 38, 156, 163] | [151, 154, 162, 164, 165] | [252, 253, 261, 265] | [172, 179] |  | [248, 249] |  |
|  | Microbicides | [188, 190, 195] |  |  |  |  |  |  |  |  |  |  |
|  | DOTS |  |  | [108, 110, 129, 130, 145, 147] |  |  |  |  |  |  |  |  |
|  | TST |  |  |  |  |  |  |  | [179-181] | [169, 170] |  |  |
|  | IPT |  |  | [107, 118, 119, 132, 135, 146, 148, 149] |  |  |  | [254, 273] |  |  |  |  |
|  | MDA |  |  |  |  |  |  | [252, 259, 262, 268] |  |  |  |  |
| **Screening** | Surveillance / screening | [199, 207, 209] |  | [109, 113, 114, 116, 117, 121, 122, 129, 130, 135, 139, 145, 147-149, 264] | [44, 47, 51-53, 57, 67, 71, 75, 76, 89, 90, 92, 93, 96, 185] | [33, 36] |  |  |  |  |  |  |
|  | Monitoring treatment response | [196, 210, 214] |  | [147] |  |  |  |  |  |  |  |  |
|  | Genotyping/ DST | [210] |  | [108, 109, 114, 116, 117, 130, 144, 145, 147, 148] |  |  |  |  |  |  |  |  |
| **Vaccine** | Vaccine |  | [106, 230, 232, 234] | [116, 147] | [73, 87, 106] | [176] | [106] | [259] |  |  |  |  |
| **Behavioral** | Education | [199] |  | [109] |  |  |  |  |  |  |  |  |
|  | Increased adherence |  |  | [143] |  |  |  |  |  |  |  |  |
|  | Reduced risk behavior |  |  |  |  |  | [154] |  |  |  |  |  |
| **None** | NA | [184, 185, 198, 201, 215] | [222, 236] | [112, 115, 120, 123, 128, 131, 136, 137, 142] | [4, 13, 48, 49, 55, 61-63, 69, 70, 78, 79, 83, 85, 97-100, 102] | [4, 6, 10, 16, 17, 19, 21, 22, 28, 37, 42, 82, 167] | [6, 150, 153, 157-159, 161] | [133, 174, 267, 270, 271] |  | [171] |  |  |

1. Fresnadillo-Martinez MJ, Garcia-Merino E, Garcia-Sanchez E, Martin-del Rey A, Rodriguez-Encinas A, Rodriguez-Sanchez G, Garcia-Sanchez JE: **Prevention of an outbreak of Acinetobacter baumannii in intensive care units: study of the efficacy of different mathematical methods**. *Revista Espanola de Quimioterapia* 2015, **28**(1):10-20.

2. Wang X, Chen Y, Zhao W, Wang Y, Song Q, Liu H, Zhao J, Han X, Hu X, Grundmann H *et al*: **A data-driven mathematical model of multi-drug resistant Acinetobacter baumannii transmission in an intensive care unit**. *Scientific reports* 2015, **5**:9478.

3. Doan TN, Kong DCM, Marshall C, Kirkpatrick CMJ, McBryde ES: **Modeling the impact of interventions against Acinetobacter baumannii transmission in intensive care units**. *Virulence* 2016, **7**(2):141-152.

4. Ballarin A, Posteraro B, Demartis G, Gervasi S, Panzarella F, Torelli R, Paroni Sterbini F, Morandotti G, Posteraro P, Ricciardi W *et al*: **Forecasting ESKAPE infections through a time-varying auto-adaptive algorithm using laboratory-based surveillance data**. *BMC Infectious Diseases* 2014, **14**(1).

5. Singer RS, Cox LA, Jr., Dickson JS, Hurd HS, Phillips I, Miller GY: **Modeling the relationship between food animal health and human foodborne illness**. *Preventive veterinary medicine* 2007, **79**(2-4):186-203.

6. van Bunnik BA, Ssematimba A, Hagenaars TJ, Nodelijk G, Haverkate MR, Bonten MJ, Hayden MK, Weinstein RA, Bootsma MC, De Jong MC: **Small distances can keep bacteria at bay for days**. *Proceedings of the National Academy of Sciences of the United States of America* 2014, **111**(9):3556-3560.

7. DalBen MF, Teixeira Mendes E, Moura ML, Abdel Rahman D, Peixoto D, Alves Dos Santos S, Barcelos de Figueiredo W, Vitale Mendes P, Utino Taniguchi L, Bezerra Coutinho FA *et al*: **A Model-Based Strategy to Control the Spread of Carbapenem-Resistant Enterobacteriaceae: Simulate and Implement**. *Infection control and hospital epidemiology* 2016, **37**(11):1315-1322.

8. McBryde ES, McElwain DLS: **A mathematical model investigating the impact of an environmental reservoir on the prevalence and control of vancomycin-resistant enterococci [3]**. *Journal of Infectious Diseases* 2006, **193**(10):1473-1474.

9. Cooper BS, Medley GF, Bradley SJ, Scott GM: **An augmented data method for the analysis of nosocomial infection data**. *American Journal of Epidemiology* 2008, **168**(5):548-557.

10. Wolkewitz M, Dettenkofer M, Bertz H, Schumacher M, Huebner J: **Statistical epidemic modeling with hospital outbreak data**. *Statistics in Medicine* 2008, **27**(30):6522-6531.

11. Ortiz AR, Banks HT, Castillo-Chavez C, Chowell G, Wang X: **A DETERMINISTIC METHODOLOGY FOR ESTIMATION OF PARAMETERS IN DYNAMIC MARKOV CHAIN MODELS**. *J Biol Syst* 2011, **19**(1):71-100.

12. Yahdi M, Abdelmageed S, Lowden J, Tannenbaum L: **Vancomycin-resistant enterococci colonization-infection model: parameter impacts and outbreak risks**. *J Biol Dyn* 2012, **6**:645-662.

13. Lee BY, Wong KF, Bartsch SM, Yilmaz SL, Avery TR, Brown ST, Song Y, Singh A, Kim DS, Huang SS: **The Regional Healthcare Ecosystem Analyst (RHEA): a simulation modeling tool to assist infectious disease control in a health system**. *J Am Med Inform Assoc* 2013, **20**(e1):e139-146.

14. Lowden J, Miller Neilan R, Yahdi M: **Optimal control of vancomycin-resistant enterococci using preventive care and treatment of infections**. *Mathematical Biosciences* 2014, **249**(1):8-17.

15. Grima DT, Webb GF, D'Agata EM: **Mathematical model of the impact of a nonantibiotic treatment for Clostridium difficile on the endemic prevalence of vancomycin-resistant enterococci in a hospital setting**. *Computational and mathematical methods in medicine* 2012, **2012**:605861.

16. Haverkate MR, Dautzenberg MJD, Ossewaarde TJM, Van Der Zee A, Den Hollander JG, Troelstra A, Bonten MJM, Bootsma MCJ: **Within-host and population transmission of blaOXA-48 in K. pneumoniae and E. coli**. *PLoS ONE* 2015, **10**(10).

17. Andraud M, Rose N, Laurentie M, Sanders P, Le Roux A, Cariolet R, Chauvin C, Jouy E: **Estimation of transmission parameters of a fluoroquinolone-resistant Escherichia coli strain between pigs in experimental conditions**. *Veterinary Research* 2011, **42**(1).

18. Kiffer CR, Camargo EC, Shimakura SE, Ribeiro PJ, Jr., Bailey TC, Pignatari AC, Monteiro AM: **A spatial approach for the epidemiology of antibiotic use and resistance in community-based studies: the emergence of urban clusters of Escherichia coli quinolone resistance in Sao Paulo, Brasil**. *International journal of health geographics* 2011, **10**:17.

19. Call DR, Matthews L, Subbiah M, Liu J: **Do antibiotic residues in soils play a role in amplification and transmission of antibiotic resistant bacteria in cattle populations?** *Frontiers in microbiology* 2013, **4**:193.

20. Volkova VV, Lu Z, Lanzas C, Grohn YT: **Evaluating targets for control of plasmid-mediated antimicrobial resistance in enteric commensals of beef cattle: a modelling approach**. *Epidemiol Infect* 2013, **141**(11):2294-2312.

21. Græsbøll K, Nielsen SS, Toft N, Christiansen LE: **How fitness reduced, antimicrobial resistant bacteria survive and spread: A multiple pig - multiple bacterial strain model**. *PLoS ONE* 2014, **9**(7).

22. Obolski U, Dellus-Gur E, Stein GY, Hadany L: **Antibiotic cross-resistance in the lab and resistance co-occurrence in the clinic: Discrepancies and implications in E.coli**. *Infection, genetics and evolution : journal of molecular epidemiology and evolutionary genetics in infectious diseases* 2016, **40**:155-161.

23. Talaminos A, Lopez-Cerero L, Calvillo J, Pascual A, Roa LM, Rodriguez-Bano J: **Modelling the epidemiology of Escherichia coli ST131 and the impact of interventions on the community and healthcare centres**. *Epidemiol Infect* 2016, **144**(9):1974-1982.

24. Domenech de Cellès M, Zahar JR, Abadie V, Guillemot D: **Limits of patient isolation measures to control extended-spectrum beta-lactamase-producing Enterobacteriaceae: model-based analysis of clinical data in a pediatric ward**. *BMC Infectious Diseases* 2013, **13**(1).

25. Pelat C, Kardas-Sloma L, Birgand G, Ruppe E, Schwarzinger M, Andremont A, Lucet JC, Yazdanpanah Y: **Hand Hygiene, Cohorting, or Antibiotic Restriction to Control Outbreaks of Multidrug-Resistant Enterobacteriaceae**. *Infect Control Hosp Epidemiol* 2016, **37**(3):272-280.

26. Boldin B, Bonten MJ, Diekmann O: **Relative effects of barrier precautions and topical antibiotics on nosocomial bacterial transmission: results of multi-compartment models**. *Bull Math Biol* 2007, **69**(7):2227-2248.

27. D'Agata EM, Magal P, Olivier D, Ruan S, Webb GF: **Modeling antibiotic resistance in hospitals: the impact of minimizing treatment duration**. *J Theor Biol* 2007, **249**(3):487-499.

28. Abatih EN, Alban L, Ersboll AK, Lo Fo Wong DM: **Impact of antimicrobial usage on the transmission dynamics of antimicrobial resistant bacteria among pigs**. *J Theor Biol* 2009, **256**(4):561-573.

29. Haber M, Levin BR, Kramarz P: **Antibiotic control of antibiotic resistance in hospitals: a simulation study**. *BMC infectious diseases* 2010, **10**:254.

30. Sun HR, Lu X, Ruan S: **Qualitative analysis of models with different treatment protocols to prevent antibiotic resistance**. *Mathematical Biosciences* 2010, **227**(1):56-67.

31. Chow K, Wang X, Curtiss R, 3rd, Castillo-Chavez C: **Evaluating the efficacy of antimicrobial cycling programmes and patient isolation on dual resistance in hospitals**. *J Biol Dyn* 2011, **5**(1):27-43.

32. Kouyos RD, Abel zur Wiesch P, Bonhoeffer S: **Informed switching strongly decreases the prevalence of antibiotic resistance in hospital wards**. *PLoS Computational Biology* 2011, **7**(3).

33. D'Agata EMC, Horn MA, Ruan S, Webb GF, Wares JR: **Efficacy of infection control interventions in reducing the spread of multidrug-resistant organisms in the hospital setting**. *PLoS ONE* 2012, **7**(2).

34. Joyner ML, Manning CC, Canter BN: **Modeling the effects of introducing a new antibiotic in a hospital setting: A case study**. *Math Biosci Eng* 2012, **9**(3):601-625.

35. Obolski U, Hadany L: **Implications of stress-induced genetic variation for minimizing multidrug resistance in bacteria**. *BMC medicine* 2012, **10**:89.

36. Cheong T, Grant JL: **A PRELIMINARY MATHEMATICAL ANALYSIS FOR UNDERSTANDING TRANSMISSION DYNAMICS OF NOSOCOMIAL INFECTIONS IN A NICU**. *Int J Ind Eng-Theory Appl Pract* 2013, **20**(3-4):241-251.

37. Levin BR, Baquero F, Johnsen PJ: **A model-guided analysis and perspective on the evolution and epidemiology of antibiotic resistance and its future**. *Current Opinion in Microbiology* 2014, **19**(1):83-89.

38. Colijn C, Cohen T: **How competition governs whether moderate or aggressive treatment minimizes antibiotic resistance**. *Elife* 2015, **4**.

39. Gao X, Pan Q, He M: **Transmission dynamics of resistant bacteria in a predator-prey system**. *Computational and Mathematical Methods in Medicine* 2015, **2015**.

40. Obolski U, Stein GY, Hadany L: **Antibiotic Restriction Might Facilitate the Emergence of Multi-drug Resistance**. *PLoS Comput Biol* 2015, **11**(6):e1004340.

41. Beams AB, Toth DJ, Khader K, Adler FR: **Harnessing Intra-Host Strain Competition to Limit Antibiotic Resistance: Mathematical Model Results**. *Bull Math Biol* 2016, **78**(9):1828-1846.

42. Qu L, Pan Q, Gao X, He M: **Population Dynamics of Patients with Bacterial Resistance in Hospital Environment**. *Computational and Mathematical Methods in Medicine* 2016, **2016**.

43. Sypsa V, Psichogiou M, Bouzala GA, Hadjihannas L, Hatzakis A, Daikos GL: **Transmission Dynamics of Carbapenemase-Producing Klebsiella Pneumoniae and Anticipated Impact of Infection Control Strategies in a Surgical Unit**. *Plos One* 2012, **7**(7):11.

44. Bootsma MCJ, Diekmann O, Bonten MJM: **Controlling methicillin-resistant Staphylococcus aureus: Quantifying the effects of interventions and rapid diagnostic testing**. *Proceedings of the National Academy of Sciences of the United States of America* 2006, **103**(14):5620-5625.

45. McBryde ES, Pettitt AN, McElwain DL: **A stochastic mathematical model of methicillin resistant Staphylococcus aureus transmission in an intensive care unit: predicting the impact of interventions**. *J Theor Biol* 2007, **245**(3):470-481.

46. Pettitt AN, Forrester ML, Gibson GJ: **Bayesian inference of hospital-acquired infectious diseases and control measures given imperfect surveillance data**. *Biostatistics* 2007, **8**(2):383-401.

47. Robotham JV, Jenkins DR, Medley GF: **Screening strategies in surveillance and control of methicillin-resistant Staphylococcus aureus (MRSA)**. *Epidemiol Infect* 2007, **135**(2):328-342.

48. Drovandi CC, Pettitt AN: **Multivariate Markov process models for the transmission of methicillin-resistant Staphylococcus aureus in a hospital ward**. *Biometrics* 2008, **64**(3):851-859.

49. Allen BD, Perla RJ: **A long-term forecast of MRSA daily burden using logistic modeling**. *Clinical laboratory science : journal of the American Society for Medical Technology* 2009, **22**(1):26-29.

50. Beggs CB, Shepherd SJ, Kerr KG: **How does healthcare worker hand hygiene behaviour impact upon the transmission of MRSA between patients?: An analysis using a Monte Carlo model**. *BMC Infectious Diseases* 2009, **9**.

51. D'Agata EM, Webb GF, Horn MA, Moellering RC, Jr., Ruan S: **Modeling the invasion of community-acquired methicillin-resistant Staphylococcus aureus into hospitals**. *Clinical infectious diseases : an official publication of the Infectious Diseases Society of America* 2009, **48**(3):274-284.

52. Skov RL, Jensen KS: **Community-associated meticillin-resistant Staphylococcus aureus as a cause of hospital-acquired infections**. *The Journal of hospital infection* 2009, **73**(4):364-370.

53. Barnes S, Golden B, Wasil E: **MRSA Transmission Reduction Using Agent-Based Modeling and Simulation**. *INFORMS J Comput* 2010, **22**(4):635-646.

54. D'Agata EM, Webb GF, Pressley J: **Rapid Emergence of Co-colonization with Community-acquired and Hospital-Acquired Methicillin-Resistant Staphylococcus aureus Strains in the Hospital Setting**. *Mathematical modelling of natural phenomena* 2010, **5**(3):76-73.

55. Donker T, Wallinga J, Grundmann H: **Patient referral patterns and the spread of hospital-acquired infections through national health care networks**. *PLoS Computational Biology* 2010, **6**(3).

56. Friedman A, Ziyadi N, Boushaba K: **A model of drug resistance with infection by health care workers**. *Math Biosci Eng* 2010, **7**(4):779-792.

57. Lee BY, Bailey RR, Smith KJ, Muder RR, Strotmeyer ES, Lewis GJ, Ufberg PJ, Song Y, Harrison LH: **Universal methicillin-resistant Staphylococcus aureus (MRSA) surveillance for adults at hospital admission: an economic model and analysis**. *Infection control and hospital epidemiology* 2010, **31**(6):598-606.

58. Pressley J, D'Agata EMC, Webb GF: **The effect of co-colonization with community-acquired and hospital-acquired methicillin-resistant Staphylococcus aureus strains on competitive exclusion**. *Journal of Theoretical Biology* 2010, **264**(3):645-656.

59. Webb GF, Horn MA, D'Agata EM, Moellering RC, Jr., Ruan S: **Competition of hospital-acquired and community-acquired methicillin-resistant Staphylococcus aureus strains in hospitals**. *J Biol Dyn* 2010, **4**(1):115-129.

60. Barnes SL, Harris AD, Golden BL, Wasil EA, Furuno JP: **Contribution of interfacility patient movement to overall methicillin-resistant Staphylococcus aureus prevalence levels**. *Infection control and hospital epidemiology* 2011, **32**(11):1073-1078.

61. Bootsma MC, Wassenberg MW, Trapman P, Bonten MJ: **The nosocomial transmission rate of animal-associated ST398 meticillin-resistant Staphylococcus aureus**. *Journal of the Royal Society, Interface* 2011, **8**(57):578-584.

62. Christopher S, Verghis RM, Antonisamy B, Sowmyanarayanan TV, Brahmadathan KN, Kang G, Cooper BS: **Transmission dynamics of methicillin-resistant Staphylococcus aureus in a medical intensive care unit in India**. *PLoS ONE* 2011, **6**(7).

63. Heller J, Innocent GT, Denwood M, Reid SWJ, Kelly L, Mellor DJ: **Assessing the probability of acquisition of meticillin-resistant Staphylococcus aureus (MRSA) in a dog using a nested stochastic simulation model and logistic regression sensitivity analysis**. *Preventive Veterinary Medicine* 2011, **99**(2-4):211-224.

64. Lee BY, McGlone SM, Wong KF, Yilmaz SL, Avery TR, Song Y, Christie R, Eubank S, Brown ST, Epstein JM *et al*: **Modeling the spread of methicillin-resistant Staphylococcus aureus (MRSA) outbreaks throughout the hospitals in Orange County, California**. *Infection control and hospital epidemiology* 2011, **32**(6):562-572.

65. Lee BY, Song Y, McGlone SM, Bailey RR, Feura JM, Tai JH, Lewis GJ, Wiringa AE, Smith KJ, Muder RR *et al*: **The economic value of screening haemodialysis patients for methicillin-resistant Staphylococcus aureus in the USA**. *Clin Microbiol Infect* 2011, **17**(11):1717-1726.

66. Milazzo L, Bown JL, Eberst A, Phillips G, Crawford JW: **Modelling of Healthcare Associated Infections: A study on the dynamics of pathogen transmission by using an individual-based approach**. *Computer Methods and Programs in Biomedicine* 2011, **104**(2):260-265.

67. Chamchod F, Ruan S: **Modeling methicillin-resistant Staphylococcus aureus in hospitals: transmission dynamics, antibiotic usage and its history**. *Theor Biol Med Model* 2012, **9**:25.

68. Chamchod F, Ruan S: **Modeling the spread of methicillin-resistant Staphylococcus aureus in nursing homes for elderly**. *PLoS ONE* 2012, **7**(1).

69. Gurieva T, Bootsma MC, Bonten MJ: **Successful Veterans Affairs initiative to prevent methicillin-resistant Staphylococcus aureus infections revisited**. *Clinical infectious diseases : an official publication of the Infectious Diseases Society of America* 2012, **54**(11):1618-1620.

70. Gurieva TV, Bootsma MCJ, Bonten MJM: **Decolonization of patients and health care workers to control nosocomial spread of methicillin-resistant Staphylococcus aureus: A simulation study**. *BMC Infectious Diseases* 2012, **12**.

71. Hall IM, Barrass I, Leach S, Pittet D, Hugonnet S: **Transmission dynamics of methicillin-resistant Staphylococcus aureus in a medical intensive care unit**. *Journal of the Royal Society, Interface* 2012, **9**(75):2639-2652.

72. Nielsen KL, Pedersen TM, Udekwu KI, Petersen A, Skov RL, Hansen LH, Hughes D, Frimodt-Moller N: **Fitness cost: a bacteriological explanation for the demise of the first international methicillin-resistant Staphylococcus aureus epidemic**. *The Journal of antimicrobial chemotherapy* 2012, **67**(6):1325-1332.

73. Tekle YI, Nielsen KM, Liu J, Pettigrew MM, Meyers LA, Galvani AP, Townsend JP: **Controlling antimicrobial resistance through targeted, vaccine-induced replacement of strains**. *PLoS One* 2012, **7**(12):e50688.

74. Wang X, Xiao Y, Wang J, Lu X: **A mathematical model of effects of environmental contamination and presence of volunteers on hospital infections in China**. *J Theor Biol* 2012, **293**:161-173.

75. Deeny SR, Cooper BS, Cookson B, Hopkins S, Robotham JV: **Targeted versus universal screening and decolonization to reduce healthcare-associated meticillin-resistant Staphylococcus aureus infection**. *Journal of Hospital Infection* 2013, **85**(1):33-44.

76. Gurieva T, Bootsma MC, Bonten MJ: **Cost and effects of different admission screening strategies to control the spread of methicillin-resistant Staphylococcus aureus**. *PLoS Comput Biol* 2013, **9**(2):e1002874.

77. Kardas-Sloma L, Boelle PY, Opatowski L, Guillemot D, Temime L: **Antibiotic reduction campaigns do not necessarily decrease bacterial resistance: the example of methicillin-resistant Staphylococcus aureus**. *Antimicrob Agents Chemother* 2013, **57**(9):4410-4416.

78. Kong F, Paterson DL, Whitby M, Coory M, Clements ACA: **A hierarchical spatial modelling approach to investigate MRSA transmission in a tertiary hospital**. *BMC Infectious Diseases* 2013, **13**(1).

79. Kouyos R, Klein E, Grenfell B: **Hospital-community interactions foster coexistence between methicillin-resistant strains of Staphylococcus aureus**. *PLoS Pathog* 2013, **9**(2):e1003134.

80. Lee BY, Bartsch SM, Wong KF, Singh A, Avery TR, Kim DS, Brown ST, Murphy CR, Yilmaz SL, Potter MA *et al*: **The importance of nursing homes in the spread of methicillin-resistant Staphylococcus aureus (MRSA) among hospitals**. *Med Care* 2013, **51**(3):205-215.

81. Lee BY, Singh A, Bartsch SM, Wong KF, Kim DS, Avery TR, Brown ST, Murphy CR, Yilmaz SL, Huang SS: **The potential regional impact of contact precaution use in nursing homes to control methicillin-resistant Staphylococcus aureus**. *Infection control and hospital epidemiology* 2013, **34**(2):151-160.

82. Lee BY, Yilmaz SL, Wong KF, Bartsch SM, Eubank S, Song Y, Avery TR, Christie R, Brown ST, Epstein JM *et al*: **Modeling the regional spread and control of vancomycin-resistant enterococci**. *Am J Infect Control* 2013, **41**(8):668-673.

83. Moxnes JF, de Blasio BF, Leegaard TM, Moen AEF: **Methicillin-Resistant Staphylococcus aureus (MRSA) Is Increasing in Norway: A Time Series Analysis of Reported MRSA and Methicillin-Sensitive S. aureus Cases, 1997-2010**. *PLoS ONE* 2013, **8**(8).

84. Plipat N, Spicknall IH, Koopman JS, Eisenberg JNS: **The dynamics of methicillin-resistant Staphylococcus aureus exposure in a hospital model and the potential for environmental intervention**. *Bmc Infectious Diseases* 2013, **13**:11.

85. Wang X, Panchanathan S, Chowell G: **A data-driven mathematical model of CA-MRSA transmission among age groups: evaluating the effect of control interventions**. *PLoS Comput Biol* 2013, **9**(11):e1003328.

86. Worby CJ, Jeyaratnam D, Robotham JV, Kypraios T, O'Neill PD, De Angelis D, French G, Cooper BS: **Estimating the effectiveness of isolation and decolonization measures in reducing transmission of methicillin-resistant Staphylococcus aureus in hospital general wards**. *Am J Epidemiol* 2013, **177**(11):1306-1313.

87. Hogea C, van Effelterre T, Acosta CJ: **A basic dynamic transmission model of Staphylococcus aureus in the US population**. *Epidemiol Infect* 2014, **142**(3):468-478.

88. Deeny SR, Worby CJ, Tosas Auguet O, Cooper BS, Edgeworth J, Cookson B, Robotham JV: **Impact of mupirocin resistance on the transmission and control of healthcare-associated MRSA**. *The Journal of antimicrobial chemotherapy* 2015, **70**(12):3366-3378.

89. Gidengil CA, Gay C, Huang SS, Platt R, Yokoe D, Lee GM: **Cost-effectiveness of strategies to prevent methicillin-resistant Staphylococcus aureus transmission and infection in an intensive care unit**. *Infection control and hospital epidemiology* 2015, **36**(1):17-27.

90. Ziakas PD, Zacharioudakis IM, Zervou FN, Mylonakis E: **Methicillin-resistant staphylococcus aureus prevention strategies in the ICU: A clinical decision analysis**. *Critical Care Medicine* 2015, **43**(2):382-393.

91. Agusto FB: **Optimal control of methicillin-resistant Staphylococcus aureus transmission in hospital settings**. *Applied Mathematical Modelling* 2016, **40**(7-8):4822-4843.

92. Berk Z, Laurenson Y, Forbes AB, Kyriazakis I: **Modelling the consequences of targeted selective treatment strategies on performance and emergence of anthelmintic resistance amongst grazing calves**. *International Journal for Parasitology-Drugs and Drug Resistance* 2016, **6**(3):258-271.

93. Ding W, Webb GF: **Optimal control applied to community-acquired methicillin-resistant Staphylococcus aureus in hospitals**. *J Biol Dyn* 2016:1-14.

94. Hetem DJ, Bootsma MCJ, Bonten MJM, Weinstein RA: **Prevention of Surgical Site Infections: Decontamination with Mupirocin Based on Preoperative Screening for Staphylococcus aureus Carriers or Universal Decontamination?** *Clinical Infectious Diseases* 2016, **62**(5):631-636.

95. López-García M: **Stochastic descriptors in an SIR epidemic model for heterogeneous individuals in small networks**. *Mathematical Biosciences* 2016, **271**:42-61.

96. Robotham JV, Deeny SR, Fuller C, Hopkins S, Cookson B, Stone S: **Cost-effectiveness of national mandatory screening of all admissions to English National Health Service hospitals for meticillin-resistant Staphylococcus aureus: A mathematical modelling study**. *The Lancet Infectious Diseases* 2016, **16**(3):348-356.

97. Worby CJ, Chang HH, Hanage WP, Lipsitch M: **The distribution of pairwise genetic distances: A tool for investigating disease transmission**. *Genetics* 2014, **198**(4):1395-1404.

98. Ciccolini M, Dahl J, Chase-Topping ME, Woolhouse ME: **Disease transmission on fragmented contact networks: livestock-associated Methicillin-resistant Staphylococcus aureus in the Danish pig-industry**. *Epidemics* 2012, **4**(4):171-178.

99. Hetem DJ, Westh H, Boye K, Jarlov JO, Bonten MJ, Bootsma MC: **Nosocomial transmission of community-associated methicillin-resistant Staphylococcus aureus in Danish Hospitals**. *The Journal of antimicrobial chemotherapy* 2012, **67**(7):1775-1780.

100. Kajita E, Okano JT, Bodine EN, Layne SP, Blower S: **Modelling an outbreak of an emerging pathogen**. *Nature Reviews Microbiology* 2007, **5**(9):700-709.

101. Wang X, Xiao Y, Wang J, Lu X: **Stochastic disease dynamics of a hospital infection model**. *Mathematical biosciences* 2013, **241**(1):115-124.

102. Hetem DJ, Bootsma MC, Troelstra A, Bonten MJ: **Transmissibility of livestock-associated methicillin-resistant Staphylococcus aureus**. *Emerging infectious diseases* 2013, **19**(11):1797-1802.

103. Kardas-Sloma L, Boelle PY, Opatowski L, Brun-Buisson C, Guillemot D, Temime L: **Impact of antibiotic exposure patterns on selection of community-associated methicillin-resistant Staphylococcus aureus in hospital settings**. *Antimicrob Agents Chemother* 2011, **55**(10):4888-4895.

104. Cooper BS, Kypraios T, Batra R, Wyncoll D, Tosas O, Edgeworth JD: **Quantifying type-specific reproduction numbers for nosocomial pathogens: Evidence for heightened transmission of an Asian sequence type 239 MRSA clone**. *PLoS Computational Biology* 2012, **8**(4).

105. Batina NG, Crnich CJ, Anderson DF, Döpfer D: **Identifyingconditions for elimination and epidemic potential of methicillin-resistant Staphylococcus aureus in nursing homes**. *Antimicrobial Resistance and Infection Control* 2016, **5**(1).

106. Joice R, Lipsitch M: **Targeting imperfect vaccines against drug-resistance determinants: a strategy for countering the rise of drug resistance**. *PLoS One* 2013, **8**(7):e68940.

107. Cohen T, Lipsitch M, Walensky RP, Murray M: **Beneficial and perverse effects of isoniazid preventive therapy for latent tuberculosis infection in HIV-tuberculosis coinfected populations**. *Proceedings of the National Academy of Sciences of the United States of America* 2006, **103**(18):7042-7047.

108. Resch SC, Salomon JA, Murray M, Weinstein MC: **Cost-effectiveness of treating multidrug-resistant tuberculosis**. *PLoS Medicine* 2006, **3**(7):1048-1057.

109. Basu S, Andrews JR, Poolman EM, Gandhi NR, Shah NS, Moll A, Moodley P, Galvani AP, Friedland GH: **Prevention of nosocomial transmission of extensively drug-resistant tuberculosis in rural South African district hospitals: an epidemiological modelling study**. *Lancet (London, England)* 2007, **370**(9597):1500-1507.

110. Rodrigues P, Gomes MG, Rebelo C: **Drug resistance in tuberculosis--a reinfection model**. *Theor Popul Biol* 2007, **71**(2):196-212.

111. Basu S, Galvani AP: **The transmission and control of XDR TB in South Africa: An operations research and mathematical modelling approach**. *Epidemiology and Infection* 2008, **136**(12):1585-1598.

112. Basu S, Orenstein E, Galvani AP: **The theoretical influence of immunity between strain groups on the progression of drug-resistant tuberculosis epidemics**. *The Journal of infectious diseases* 2008, **198**(10):1502-1513.

113. Cohen T, Colijn C, Finklea B, Wright A, Zignol M, Pym A, Murray M: **Are survey-based estimates of the burden of drug resistant TB too low? Insight from a simulation study**. *PLoS ONE* 2008, **3**(6).

114. Dowdy DW, Chaisson RE, Maartens G, Corbett EL, Dorman SE: **Impact of enhanced tuberculosis diagnosis in South Africa: a mathematical model of expanded culture and drug susceptibility testing**. *Proceedings of the National Academy of Sciences of the United States of America* 2008, **105**(32):11293-11298.

115. Gumel AB, Song B: **Existence of multiple-stable equilibria for a multi-drug-resistant model of mycobacterium tuberculosis**. *Mathematical Biosciences and Engineering* 2008, **5**(3):437-455.

116. Abu-Raddad LJ, Sabatelli L, Achterberg JT, Sugimoto JD, Longini IM, Jr., Dye C, Halloran ME: **Epidemiological benefits of more-effective tuberculosis vaccines, drugs, and diagnostics**. *Proceedings of the National Academy of Sciences of the United States of America* 2009, **106**(33):13980-13985.

117. Basu S, Frledland GH, Medlock J, Andrews JR, Shah NS, Gandhi NR, Moll A, Moodley P, Sturm AW, Galvani AP: **Averting epidemics of extensively drug-resistant tuberculosis**. *Proceedings of the National Academy of Sciences of the United States of America* 2009, **106**(18):7672-7677.

118. Basu S, Maru D, Poolman E, Galvani A: **Primary and secondary tuberculosis preventive treatment in HIV clinics: simulating alternative strategies**. *The international journal of tuberculosis and lung disease : the official journal of the International Union against Tuberculosis and Lung Disease* 2009, **13**(5):652-658.

119. Colijn C, Cohen T, Murray M: **Latent coinfection and the maintenance of strain diversity**. *Bull Math Biol* 2009, **71**(1):247-263.

120. Luciani F, Sisson SA, Jiang H, Francis AR, Tanaka MM: **The epidemiological fitness cost of drug resistance in Mycobacterium tuberculosis**. *Proceedings of the National Academy of Sciences of the United States of America* 2009, **106**(34):14711-14715.

121. Uys PW, Warren R, van Helden PD, Murray M, Victor TC: **Potential of rapid diagnosis for controlling drug-susceptible and drug-resistant tuberculosis in communities where Mycobacterium tuberculosis infections are highly prevalent**. *Journal of clinical microbiology* 2009, **47**(5):1484-1490.

122. Cohen T, Hedt BL, Pagano M: **Estimating the magnitude and direction of bias in tuberculosis drug resistance surveys conducted only in the public sector: a simulation study**. *BMC Public Health* 2010, **10**:355.

123. Jacob BG, Krapp F, Ponce M, Gotuzzo E, Griffith DA, Novak RJ: **Accounting for autocorrelation in multi-drug resistant tuberculosis predictors using a set of parsimonious orthogonal eigenvectors aggregated in geographic space**. *Geospatial Health* 2010, **4**(2):201-217.

124. Oxlade O, Schwartzman K, Pai M, Heymann J, Benedetti A, Royce S, Menzies D: **Predicting outcomes and drug resistance with standardised treatment of active tuberculosis**. *The European respiratory journal* 2010, **36**(4):870-877.

125. Bhunu CP: **Mathematical analysis of a three-strain tuberculosis transmission model**. *Applied Mathematical Modelling* 2011, **35**(9):4647-4660.

126. De Espíndola AL, Bauch CT, Troca Cabella BC, Martinez AS: **An agent-based computational model of the spread of tuberculosis**. *Journal of Statistical Mechanics: Theory and Experiment* 2011, **2011**(5).

127. Liu YQ, Sun ZD, Sun GQ, Zhong Q, Jiang L, Zhou L, Qiao YP, Jia ZW: **Modeling Transmission of Tuberculosis with MDR and Undetected Cases**. *Discrete Dynamics in Nature and Society* 2011:12.

128. Sergeev R, Colijn C, Cohen T: **Models to understand the population-level impact of mixed strain M. tuberculosis infections**. *Journal of Theoretical Biology* 2011, **280**(1):88-100.

129. Thomas EG, Barrington HE, Lokuge KM, Mercer GN: **Modelling the spread of tuberculosis, including drug resistance and hiv: A case study in Papua New Guinea's western province**. *ANZIAM Journal* 2011, **52**(1):26-45.

130. Liao CM, Lin YJ: **Assessing the transmission risk of multidrug-resistant Mycobacterium tuberculosis epidemics in regions of Taiwan**. *International Journal of Infectious Diseases* 2012, **16**(10):e739-e747.

131. Sergeev R, Colijn C, Murray M, Cohen T: **Modeling the dynamic relationship between HIV and the risk of drug-resistant tuberculosis**. *Science Translational Medicine* 2012, **4**(135).

132. Mills HL, Cohen T, Colijn C: **Community-wide isoniazid preventive therapy drives drug-resistant tuberculosis: A model-based analysis**. *Science Translational Medicine* 2013, **5**(180).

133. Agusto FB: **Malaria Drug Resistance: The Impact of Human Movement and Spatial Heterogeneity**. *Bull Math Biol* 2014, **76**(7):1607-1641.

134. Ahmadin, Fatmawati: **Mathematical modeling of drug resistance in tuberculosis transmission and optimal control treatment**. *Applied Mathematical Sciences* 2014, **8**(92):4547-4559.

135. Denholm JT, McBryde ES: **Can Australia eliminate TB? Modelling immigration strategies for reaching MDG targets in a low-transmission setting**. *Australian and New Zealand journal of public health* 2014, **38**(1):78-82.

136. Lin YJ, Liao CM: **Seasonal dynamics of tuberculosis epidemics and implications for multidrug-resistant infection risk assessment**. *Epidemiology and Infection* 2014, **142**(2):358-370.

137. Raimundo SM, Yang HM, Venturino E: **Theoretical assessment of the relative incidences of sensitive and resistant tuberculosis epidemic in presence of drug treatment**. *Mathematical Biosciences and Engineering* 2014, **11**(4):971-993.

138. Shrestha S, Knight GM, Fofana M, Cohen T, White RG, Cobelens F, Dowdy DW: **Drivers and trajectories of resistance to new first-line drug regimens for tuberculosis**. *Open forum infectious diseases* 2014, **1**(2):ofu073.

139. Trauer JM, Denholm JT, McBryde ES: **Construction of a mathematical model for tuberculosis transmission in highly endemic regions of the Asia-Pacific**. *J Theor Biol* 2014, **358**:74-84.

140. Agusto FB, Cook J, Shelton PD, Wickers MG: **Mathematical Model of MDR-TB and XDR-TB with Isolation and Lost to Follow-Up**. *Abstract and Applied Analysis* 2015, **2015**.

141. Kendall EA, Fofana MO, Dowdy DW: **Burden of transmitted multidrug resistance in epidemics of tuberculosis: A transmission modelling analysis**. *The Lancet Respiratory Medicine* 2015, **3**(12):963-972.

142. Knight GM, Colijn C, Shrestha S, Fofana M, Cobelens F, White RG, Dowdy DW, Cohen T: **The Distribution of Fitness Costs of Resistance-Conferring Mutations Is a Key Determinant for the Future Burden of Drug-Resistant Tuberculosis: A Model-Based Analysis**. *Clinical Infectious Diseases* 2015, **61**:S147-S154.

143. Lin HH, Wang L, Zhang H, Ruan Y, Chin DP, Dye C: **Tuberculosis control in China: use of modelling to develop targets and policies**. *Bulletin of the World Health Organization* 2015, **93**(11):790-798.

144. Sachdeva KS, Raizada N, Gupta RS, Nair SA, Denkinger C, Paramasivan CN, Kulsange S, Thakur R, Dewan P, Boehme C *et al*: **The Potential Impact of Up-Front Drug Sensitivity Testing on India's Epidemic of Multi-Drug Resistant Tuberculosis**. *PLoS One* 2015, **10**(7):e0131438.

145. Suen SC, Bendavid E, Goldhaber-Fiebert JD: **Cost-effectiveness of improvements in diagnosis and treatment accessibility for tuberculosis control in India**. *International Journal of Tuberculosis and Lung Disease* 2015, **19**(9):1115-1124.

146. Kunkel A, Crawford FW, Shepherd J, Cohen T: **Benefits of continuous isoniazid preventive therapy may outweigh resistance risks in a declining tuberculosis/HIV coepidemic**. *Aids* 2016, **30**(17):2715-2723.

147. Trauer JM, Denholm JT, Waseem S, Ragonnet R, McBryde ES: **Scenario Analysis for Programmatic Tuberculosis Control in Western Province, Papua New Guinea**. *Am J Epidemiol* 2016, **183**(12):1138-1148.

148. Gilbert JA, Long EF, Brooks RP, Friedland GH, Moll AP, Townsend JP, Galvani AP, Shenoi SV: **Integrating Community-Based Interventions to Reverse the Convergent TB/HIV Epidemics in Rural South Africa**. *PLoS One* 2015, **10**(5):e0126267.

149. Gilbert JA, Shenoi SV, Moll AP, Friedland GH, Paltiel AD, Galvani AP: **Cost-effectiveness of community-based TB/HIV screening and linkage to care in rural South Africa**. *PLoS ONE* 2016, **11**(12).

150. Handel A, Regoes RR, Antia R: **The role of compensatory mutations in the emergence of drug resistance**. *PLoS Comput Biol* 2006, **2**(10):e137.

151. Chan CH, McCabe CJ, Fisman DN: **Core groups, antimicrobial resistance and rebound in gonorrhoea in North America**. *Sexually transmitted infections* 2012, **88**(3):200-204.

152. Hui BB, Ryder N, Su JY, Ward J, Chen MY, Donovan B, Fairley CK, Guy RJ, Lahra MM, Law MG *et al*: **Exploring the Benefits of Molecular Testing for Gonorrhoea Antibiotic Resistance Surveillance in Remote Settings**. *PLoS One* 2015, **10**(7):e0133202.

153. Trecker MA, Hogan DJ, Waldner CL, Dillon JA, Osgood ND: **Revised simulation model does not predict rebound in gonorrhoea prevalence where core groups are treated in the presence of antimicrobial resistance**. *Sexually transmitted infections* 2015, **91**(4):300-302.

154. Xiridou M, Soetens LC, Koedijk FD, MA VDS, Wallinga J: **Public health measures to control the spread of antimicrobial resistance in Neisseria gonorrhoeae in men who have sex with men**. *Epidemiol Infect* 2015, **143**(8):1575-1584.

155. Fingerhuth SM, Bonhoeffer S, Low N, Althaus CL: **Antibiotic-Resistant Neisseria gonorrhoeae Spread Faster with More Treatment, Not More Sexual Partners**. *PLoS Pathogens* 2016, **12**(5).

156. Hurford A, Morris AM, Fisman DN, Wu J: **Linking antimicrobial prescribing to antimicrobial resistance in the ICU: before and after an antimicrobial stewardship program**. *Epidemics* 2012, **4**(4):203-210.

157. Hald T, Lo Fo Wong DM, Aarestrup FM: **The attribution of human infections with antimicrobial resistant Salmonella bacteria in Denmark to sources of animal origin**. *Foodborne pathogens and disease* 2007, **4**(3):313-326.

158. Pitzer VE, Feasey NA, Msefula C, Mallewa J, Kennedy N, Dube Q, Denis B, Gordon MA, Heyderman RS: **Mathematical Modeling to Assess the Drivers of the Recent Emergence of Typhoid Fever in Blantyre, Malawi**. *Clinical infectious diseases : an official publication of the Infectious Diseases Society of America* 2015, **61 Suppl 4**:S251-258.

159. Stelling J, Yih WK, Galas M, Kulldorff M, Pichel M, Terragno R, Tuduri E, Espetxe S, Binsztein N, O'Brien TF *et al*: **Automated use of WHONET and SaTScan to detect outbreaks of Shigella spp. using antimicrobial resistance phenotypes**. *Epidemiol Infect* 2010, **138**(6):873-883.

160. Maher MC, Alemayehu W, Lakew T, Gaynor BD, Haug S, Cevallos V, Keenan JD, Lietman TM, Porco TC: **The fitness cost of antibiotic resistance in streptococcus pneumoniae: Insight from the field**. *PLoS ONE* 2012, **7**(1).

161. Geli P, Rolfhamre P, Almeida J, Ekdahl K: **Modeling pneumococcal resistance to penicillin in southern Sweden using artificial neural networks**. *Microbial Drug Resistance* 2006, **12**(3):149-157.

162. Wang YC, Lipsitch M: **Upgrading antibiotic use within a class: Tradeoff between resistance and treatment success**. *Proceedings of the National Academy of Sciences of the United States of America* 2006, **103**(25):9655-9660.

163. Opatowski L, Temime L, Varon E, Leclercq R, Drugeon H, Boelle PY, Guillemot D: **Antibiotic innovation may contribute to slowing the dissemination of multiresistant Streptococcus pneumoniae: the example of ketolides**. *PLoS One* 2008, **3**(5):e2089.

164. Opatowski L, Mandel J, Varon E, Boelle PY, Temime L, Guillemot D: **Antibiotic dose impact on resistance selection in the community: a mathematical model of beta-lactams and Streptococcus pneumoniae dynamics**. *Antimicrob Agents Chemother* 2010, **54**(6):2330-2337.

165. Domenech de Celles M, Opatowski L, Salomon J, Varon E, Carbon C, Boelle PY, Guillemot D: **Intrinsic epidemicity of Streptococcus pneumoniae depends on strain serotype and antibiotic susceptibility pattern**. *Antimicrob Agents Chemother* 2011, **55**(11):5255-5261.

166. Opatowski L, Varon E, Dupont C, Temime L, van der Werf S, Gutmann L, Boelle PY, Watier L, Guillemot D: **Assessing pneumococcal meningitis association with viral respiratory infections and antibiotics: insights from statistical and mathematical models**. *Proceedings Biological sciences* 2013, **280**(1764):20130519.

167. Boëlle PY, Thomas G: **Resistance to antibiotics: limit theorems for a stochastic SIS model structured by level of resistance**. *Journal of mathematical biology* 2016, **73**(6-7):1353-1378.

168. Gao D, Lietman TM, Porco TC: **Antibiotic resistance as collateral damage: The tragedy of the commons in a two-disease setting**. *Mathematical Biosciences* 2015, **263**:121-132.

169. Xu J, Vidyashankar A, Nielsen MK: **Drug Resistance or Re-Emergence ? Simulating Equine Parasites**. *ACM Trans Model Comput Simul* 2014, **24**(4):23.

170. Park AW, Haven J, Kaplan R, Gandon S: **Refugia and the evolutionary epidemiology of drug resistance**. *Biology letters* 2015, **11**(11).

171. Stauch A, Duerr HP, Dujardin JC, Vanaerschot M, Sundar S, Eichner M: **Treatment of Visceral Leishmaniasis: Model-Based Analyses on the Spread of Antimony-Resistant L. donovani in Bihar, India**. *PLoS Neglected Tropical Diseases* 2012, **6**(12).

172. Gaba S, Cabaret J, Ginot V, Silvestre A: **The early drug selection of nematodes to anthelmintics: Stochastic transmission and population in refuge**. *Parasitology* 2006, **133**(3):345-356.

173. Yakob L, Williams GM, Gray DJ, Halton K, Solon JA, Clements AC: **Slaving and release in co-infection control**. *Parasites and Vectors* 2013, **6**(1).

174. Hansen J, Day T: **Coinfection and the evolution of drug resistance**. *J Evol Biol* 2014, **27**(12):2595-2604.

175. Colijn C, Cohen T, Fraser C, Hanage W, Goldstein E, Givon-Lavi N, Dagan R, Lipsitch M: **What is the mechanism for persistent coexistence of drug-susceptible and drug-resistant strains of Streptococcus pneumoniae?** *Journal of the Royal Society, Interface* 2010, **7**(47):905-919.

176. Mitchell PK, Lipsitch M, Hanage WP: **Carriage burden, multiple colonization and antibiotic pressure promote emergence of resistant vaccine escape pneumococci**. *Philosophical transactions of the Royal Society of London Series B, Biological sciences* 2015, **370**(1670):20140342.

177. Xu D, Sandland GJ, Minchella DJ, Feng Z: **Interactions among virulence, coinfection and drug resistance in a complex life-cycle parasite**. *J Theor Biol* 2012, **304**:197-210.

178. Yang Y, Feng Z, Xu D, Sandland GJ, Minchella DJ: **Evolution of host resistance to parasite infection in the snail-schistosome-human system**. *Journal of mathematical biology* 2012, **65**(2):201-236.

179. Leathwick DM, Waghorn TS, Miller CM, Candy PM, Oliver AM: **Managing anthelmintic resistance--use of a combination anthelmintic and leaving some lambs untreated to slow the development of resistance to ivermectin**. *Vet Parasitol* 2012, **187**(1-2):285-294.

180. Laurenson YC, Bishop SC, Forbes AB, Kyriazakis I: **Modelling the short- and long-term impacts of drenching frequency and targeted selective treatment on the performance of grazing lambs and the emergence of anthelmintic resistance**. *Parasitology* 2013, **140**(6):780-791.

181. Laurenson YCSM, Kahn LP, Bishop SC, Kyriazakis I: **Which is the best phenotypic trait for use in a targeted selective treatment strategy for growing lambs in temperate climates?** *Veterinary Parasitology* 2016, **226**:174-188.

182. Schwab AE, Churcher TS, Schwab AJ, Basanez MG, Prichard RK: **Population genetics of concurrent selection with albendazole and ivermectin or diethylcarbamazine on the possible spread of albendazole resistance in Wuchereria bancrofti**. *Parasitology* 2006, **133**:589-601.

183. Baggaley RF, Garnett GP, Ferguson NM: **Modelling the impact of antiretroviral use in resource-poor settings**. *PLoS Medicine* 2006, **3**(4):493-504.

184. Sanchez MS, Grant RM, Porco TC, Getz WM: **HIV drug-resistant strains as epidemiologic sentinels**. *Emerging Infectious Diseases* 2006, **12**(2):191-197.

185. Vardavas R, Blower S: **The emergence of HIV transmitted resistance in Botswana: "When will the WHO detection threshold be exceeded?"**. *PLoS ONE* 2007, **2**(1).

186. Lima VD, Johnston K, Hogg RS, Levy AR, Harrigan PR, Anema A, Montaner JS: **Expanded access to highly active antiretroviral therapy: a potentially powerful strategy to curb the growth of the HIV epidemic**. *The Journal of infectious diseases* 2008, **198**(1):59-67.

187. Sharomi O, Gumel AB: **Dynamical analysis of a multi-strain model of HIV in the presence of anti-retroviral drugs**. *Journal of biological dynamics* 2008, **2**(3):323-345.

188. Wilson DP, Coplan PM, Wainberg MA, Blower SM: **The paradoxical effects of using antiretroviral-based microbicides to control HIV epidemics**. *Proceedings of the National Academy of Sciences of the United States of America* 2008, **105**(28):9835-9840.

189. Bhunu CP, Garira W, Magombedze G: **Mathematical analysis of a two strain HIV/AIDS model with antiretroviral treatment**. *Acta Biotheoretica* 2009, **57**(3):361-381.

190. Dimitrov DT, Masse B, Boily MC: **Who will Benefit from a Wide-Scale Introduction of Vaginal Microbicides in Developing Countries?** *Statistical communications in infectious diseases* 2010, **2**(1):1012.

191. Hoare A, Kerr SJ, Ruxrungtham K, Ananworanich J, Law MG, Cooper DA, Phanuphak P, Wilson DP: **Hidden drug resistant HIV to emerge in the era of universal treatment access in Southeast Asia**. *PLoS One* 2010, **5**(6):e10981.

192. Marks AJ, Pillay D, McLean AR: **The effect of intrinsic stochasticity on transmitted HIV drug resistance patterns**. *J Theor Biol* 2010, **262**(1):1-13.

193. Supervie V, Garcia-Lerma JG, Heneine W, Blower S: **HIV, transmitted drug resistance, and the paradox of preexposure prophylaxis**. *Proceedings of the National Academy of Sciences of the United States of America* 2010, **107**(27):12381-12386.

194. Abbas UL, Hood G, Wetzel AW, Mellors JW: **Factors influencing the emergence and spread of HIV drug resistance arising from rollout of antiretroviral pre-exposure prophylaxis (PrEP)**. *PLoS ONE* 2011, **6**(4).

195. Dimitrov DT, Boily MC, Baggaley RF, Masse B: **Modeling the gender-specific impact of vaginal microbicides on HIV transmission**. *J Theor Biol* 2011, **288**:9-20.

196. Phillips AN, Pillay D, Garnett G, Bennett D, Vitoria M, Cambiano V, Lundgren J: **Effect on transmission of HIV-1 resistance of timing of implementation of viral load monitoring to determine switches from first to second-line antiretroviral regimens in resource-limited settings**. *AIDS* 2011, **25**(6):843-850.

197. Supervie V, Barrett M, Kahn JS, Musuka G, Moeti TL, Busang L, Blower S: **Modeling dynamic interactions between pre-exposure prophylaxis interventions & treatment programs: Predicting HIV transmission & resistance**. *Scientific Reports* 2011, **1**.

198. Li Q, Cao S, Chen X, Sun G, Liu Y, Jia Z: **Stability analysis of an HIV/AIDS dynamics model with drug resistance**. *Discrete Dynamics in Nature and Society* 2012, **2012**.

199. Lou J, Bu L, Han E, Ruan Y, Xing H, Shao Y: **Modeling primary and secondary drug resistances under China's "four-free-one-care policy"**. *International Journal of Biomathematics* 2012, **5**(5).

200. von Wyl V, Cambiano V, Jordan MR, Bertagnolio S, Miners A, Pillay D, Lundgren J, Phillips AN: **Cost-effectiveness of tenofovir instead of zidovudine for use in first-line antiretroviral therapy in settings without virological monitoring**. *PLoS ONE* 2012, **7**(8).

201. Wagner BG, Garcia-Lerma JG, Blower S: **Factors limiting the transmission of HIV mutations conferring drug resistance: fitness costs and genetic bottlenecks**. *Scientific reports* 2012, **2**:320.

202. Abbas UL, Glaubius R, Mubayi A, Hood G, Mellors JW: **Antiretroviral therapy and pre-exposure prophylaxis: combined impact on HIV transmission and drug resistance in South Africa**. *The Journal of infectious diseases* 2013, **208**(2):224-234.

203. Cambiano V, Bertagnolio S, Jordan MR, Lundgren JD, Phillips A: **Transmission of drug resistant HIV and its potential impact on mortality and treatment outcomes in resource-limited settings**. *The Journal of infectious diseases* 2013, **207 Suppl 2**:S57-62.

204. Dimitrov D, Boily MC, Brown ER, Hallett TB: **Analytic review of modeling studies of ARV based PrEP interventions reveals strong influence of drug-resistance assumptions on the population-level effectiveness**. *PLoS ONE* 2013, **8**(11).

205. Nichols BE, Boucher CA, van Dijk JH, Thuma PE, Nouwen JL, Baltussen R, van de Wijgert J, Sloot PM, van de Vijver DA: **Cost-effectiveness of pre-exposure prophylaxis (PrEP) in preventing HIV-1 infections in rural Zambia: a modeling study**. *PLoS One* 2013, **8**(3):e59549.

206. Saenz RA, Bonhoeffer S: **Nested model reveals potential amplification of an HIV epidemic due to drug resistance**. *Epidemics* 2013, **5**(1):34-43.

207. Sood N, Wagner Z, Jaycocks A, Drabo E, Vardavas R: **Test-and-treat in Los Angeles: a mathematical model of the effects of test-and-treat for the population of men who have sex with men in Los Angeles County**. *Clinical infectious diseases : an official publication of the Infectious Diseases Society of America* 2013, **56**(12):1789-1796.

208. Vijver DAMCVD, Nichols BE, Abbas UL, Boucher CAB, Cambiano V, Eaton JW, Glaubius R, Lythgoe K, Mellors J, Phillips A *et al*: **Preexposure prophylaxis will have a limited impact on HIV-1 drug resistance in sub-Saharan Africa: A comparison of mathematical models**. *AIDS* 2013, **27**(18):2943-2951.

209. Cambiano V, Bertagnolio S, Jordan MR, Pillay D, Perriëns JH, Venter F, Lundgren J, Phillips A: **Predicted levels of HIV drug resistance: Potential impact of expanding diagnosis, retention, and eligibility criteria for antiretroviral therapy initiation**. *AIDS* 2014, **28**(SUPPL. 1):S15-S23.

210. Nichols BE, Sigaloff KC, Kityo C, Hamers RL, Baltussen R, Bertagnolio S, Jordan MR, Hallett TB, Boucher CA, de Wit TF *et al*: **Increasing the use of second-line therapy is a cost-effective approach to prevent the spread of drug-resistant HIV: a mathematical modelling study**. *Journal of the International AIDS Society* 2014, **17**:19164.

211. Nichols BE, Sigaloff KC, Kityo C, Mandaliya K, Hamers RL, Bertagnolio S, Jordan MR, Boucher CA, Rinke de Wit TF, van de Vijver DA: **Averted HIV infections due to expanded antiretroviral treatment eligibility offsets risk of transmitted drug resistance: a modeling study**. *Aids* 2014, **28**(1):73-83.

212. Dimitrov DT, Boily MC, Hallett TB, Albert J, Boucher C, Mellors JW, Pillay D, van de Vijver DA: **How Much Do We Know about Drug Resistance Due to PrEP Use? Analysis of Experts' Opinion and Its Influence on the Projected Public Health Impact**. *PLoS One* 2016, **11**(7):e0158620.

213. Glaubius RL, Parikh UM, Hood G, Penrose KJ, Bendavid E, Mellors JW, Abbas UL: **Deciphering the Effects of Injectable Pre-exposure Prophylaxis for Combination Human Immunodeficiency Virus Prevention**. *Open forum infectious diseases* 2016, **3**(3):ofw125.

214. Pham QD, Wilson DP, Nguyen TV, Do NT, Truong LX, Nguyen LT, Zhang L: **Projecting the epidemiological effect, cost-effectiveness and transmission of HIV drug resistance in Vietnam associated with viral load monitoring strategies**. *The Journal of antimicrobial chemotherapy* 2016, **71**(5):1367-1379.

215. Sun X, Xiao Y, Peng Z: **Modelling HIV superinfection among men who have sex with men**. *Math Biosci Eng* 2016, **13**(1):171-191.

216. Sun X, Xiao Y, Tang S, Peng Z, Wu J, Wang N: **Early HAART Initiation May Not Reduce Actual Reproduction Number and Prevalence of MSM Infection: Perspectives from Coupled within- and between-Host Modelling Studies of Chinese MSM Populations**. *PLoS ONE* 2016, **11**(3).

217. Regoes RR, Bonhoeffer S: **Emergence of drug-resistant influenza virus: Population dynamical considerations**. *Science* 2006, **312**(5772):389-391.

218. Alexander ME, Bowman CS, Feng Z, Gardam M, Moghadas SM, Rost G, Wu J, Yan P: **Emergence of drug resistance: implications for antiviral control of pandemic influenza**. *Proceedings Biological sciences* 2007, **274**(1619):1675-1684.

219. Debarre F, Bonhoeffer S, Regoes RR: **The effect of population structure on the emergence of drug resistance during influenza pandemics**. *Journal of the Royal Society, Interface* 2007, **4**(16):893-906.

220. Lipsitch M, Cohen T, Murray M, Levin BR: **Antiviral resistance and the control of pandemic influenza**. *PLoS Medicine* 2007, **4**(1):0111-0121.

221. Xu Y, Allen LJ, Perelson AS: **Stochastic model of an influenza epidemic with drug resistance**. *J Theor Biol* 2007, **248**(1):179-193.

222. Brockmann SO, Schwehm M, Duerr HP, Witschi M, Koch D, Vidondo B, Eichner M: **Modeling the effects of drug resistant influenza virus in a pandemic**. *Virology Journal* 2008, **5**.

223. McCaw JM, Wood JG, McCaw CT, McVernon J: **Impact of emerging antiviral drug resistance on influenza containment and spread: influence of subclinical infection and strategic use of a stockpile containing one or two drugs**. *PLoS One* 2008, **3**(6):e2362.

224. Moghadas SM: **Management of drug resistance in the population: influenza as a case study**. *Proceedings Biological sciences* 2008, **275**(1639):1163-1169.

225. Moghadas SM, Bowman CS, Rost G, Wu J: **Population-wide emergence of antiviral resistance during pandemic influenza**. *PLoS One* 2008, **3**(3):e1839.

226. Alexander ME, Dietrich SM, Hua Y, Moghadas SM: **A comparative evaluation of modelling strategies for the effect of treatment and host interactions on the spread of drug resistance**. *Journal of Theoretical Biology* 2009, **259**(2):253-263.

227. Arino J, Bowman CS, Moghadas SM: **Antiviral resistance during pandemic influenza: implications for stockpiling and drug use**. *BMC infectious diseases* 2009, **9**:8.

228. Handel A, Longini IM, Jr., Antia R: **Antiviral resistance and the control of pandemic influenza: the roles of stochasticity, evolution and model details**. *J Theor Biol* 2009, **256**(1):117-125.

229. Handel A, Longini IM, Jr., Antia R: **Intervention strategies for an influenza pandemic taking into account secondary bacterial infections**. *Epidemics* 2009, **1**(3):185-195.

230. Iwani S, Suzuki T, Takeuchi Y: **Paradox of Vaccination: Is Vaccination really effective against avian flu epidemics?** *PLoS ONE* 2009, **4**(3).

231. Moghadas SM, Bowman CS, Rost G, Fisman DN, Wu J: **Post-exposure prophylaxis during pandemic outbreaks**. *BMC medicine* 2009, **7**:73.

232. Van Den Dool C, Hak E, Bonten MJM, Wallinga J: **A model-based assessment of oseltamivir prophylaxis strategies to prevent influenza in nursing homes**. *Emerging Infectious Diseases* 2009, **15**(10):1547-1555.

233. Wu JT, Leung GM, Lipsitch M, Cooper BS, Riley S: **Hedging against antiviral resistance during the next influenza pandemic using small stockpiles of an alternative chemotherapy**. *PLoS medicine* 2009, **6**(5):e1000085.

234. Qiu Z, Feng Z: **Transmission dynamics of an influenza model with vaccination and antiviral treatment**. *Bull Math Biol* 2010, **72**(1):1-33.

235. Shim E, Chapman GB, Galvani AP: **Decision Making with Regard to Antiviral Intervention during an Influenza Pandemic**. *Med Decis Mak* 2010, **30**(4):E64-E81.

236. McCaw JM, Arinaminpathy N, Hurt AC, McVernon J, McLean AR: **A mathematical framework for estimating pathogen transmission fitness and inoculum size using data from a competitive mixtures animal model**. *PLoS Computational Biology* 2011, **7**(4).

237. Wessel L, Hua Y, Wu J, Moghadas SM: **Public health interventions for epidemics: Implications for multiple infection waves**. *BMC Public Health* 2011, **11**(SUPPL. 1).

238. Chao DL, Bloom JD, Kochin BF, Antia R, Longini Jr IM: **The global spread of drug-resistant influenza**. *Journal of the Royal Society Interface* 2012, **9**(69):648-656.

239. Dafilis MP, Moss R, McVernon J, McCaw J: **Drivers and consequences of influenza antiviral resistant-strain emergence in a capacity-constrained pandemic response**. *Epidemics* 2012, **4**(4):219-226.

240. Althouse BM, Patterson-Lomba O, Goerg GM, Hébert-Dufresne L: **The Timing and Targeting of Treatment in Influenza Pandemics Influences the Emergence of Resistance in Structured Populations**. *PLoS Computational Biology* 2013, **9**(2).

241. Chao DL: **Modeling the global transmission of antiviral-resistant influenza viruses**. *Influenza and other respiratory viruses* 2013, **7 Suppl 1**:58-62.

242. Jaberi-Douraki M, Heffernan JM, Wu J, Moghadas SM: **Optimal Treatment Profile During an Influenza Epidemic**. *Differential Equations and Dynamical Systems* 2013, **21**(3):237-252.

243. Jaberi-Douraki M, Moghadas SM: **Optimality of a time-dependent treatment profile during an epidemic**. *J Biol Dyn* 2013, **7**:133-147.

244. Patterson-Lomba O, Althouse BM, Goerg GM, Hebert-Dufresne L: **Optimizing treatment regimes to hinder antiviral resistance in influenza across time scales**. *PLoS One* 2013, **8**(3):e59529.

245. Robinson M, Stilianakis NI: **A model for the emergence of drug resistance in the presence of asymptomatic infections**. *Mathematical biosciences* 2013, **243**(2):163-177.

246. Hobbelen PHF, Paveley ND, Fraaije BA, Lucas JA, van den Bosch F: **Derivation and testing of a model to predict selection for fungicide resistance**. *Plant Pathology* 2011, **60**(2):304-313.

247. Hobbelen PHF, Paveley ND, van den Bosch F: **Delaying Selection for Fungicide Insensitivity by Mixing Fungicides at a Low and High Risk of Resistance Development: A Modeling Analysis**. *Phytopathology* 2011, **101**(10):1224-1233.

248. Hobbelen PH, Paveley ND, Oliver RP, van den Bosch F: **The usefulness of fungicide mixtures and alternation for delaying the selection for resistance in populations of Mycosphaerella graminicola on winter wheat: a modeling analysis**. *Phytopathology* 2013, **103**(7):690-707.

249. van den Berg F, van den Bosch F, Paveley ND: **Optimal Fungicide Application Timings for Disease Control Are Also an Effective Anti-Resistance Strategy: A Case Study for Zymoseptoria tritici (Mycosphaerella graminicola) on Wheat**. *Phytopathology* 2013, **103**(12):1209-1219.

250. Kitchen JL, van den Bosch F, Paveley ND, Helps J, van den Berg F: **The Evolution of Fungicide Resistance Resulting from Combinations of Foliar-Acting Systemic Seed Treatments and Foliar-Applied Fungicides: A Modeling Analysis**. *PLoS One* 2016, **11**(8):e0161887.

251. Kunkel A, Colijn C, Lipsitch M, Cohen T: **How could preventive therapy affect the prevalence of drug resistance? Causes and consequences**. *Philosophical transactions of the Royal Society of London Series B, Biological sciences* 2015, **370**(1670):20140306.

252. Maude RJ, Pontavornpinyo W, Saralamba S, Aguas R, Yeung S, Dondorp AM, Day NP, White NJ, White LJ: **The last man standing is the most resistant: Eliminating artemisinin-resistant malaria in Cambodia**. *Malaria Journal* 2009, **8**(1).

253. Laxminarayan R, Over M, Smith DL: **Will a global subsidy of new antimalarials delay the emergence of resistance and save lives?** *Health Affairs* 2006, **25**(2):325-336.

254. O'Meara WP, Smith DL, McKenzie FE: **Potential impact of intermittent preventive treatment (IPT) on spread of drug-resistant malaria**. *PLoS Medicine* 2006, **3**(5):633-642.

255. Pongtavornpinyo W, Yeung S, Hastings IM, Dondorp AM, Day NP, White NJ: **Spread of anti-malarial drug resistance: mathematical model with implications for ACT drug policies**. *Malaria journal* 2008, **7**:229.

256. Chiyaka C, Garira W, Dube S: **Effects of treatment and drug resistance on the transmission dynamics of malaria in endemic areas**. *Theor Popul Biol* 2009, **75**(1):14-29.

257. Esteva L, Gumel AB, de León CV: **Qualitative study of transmission dynamics of drug-resistant malaria**. *Mathematical and Computer Modelling* 2009, **50**(3-4):611-630.

258. Tasman H, Soewono E, Sidarto KA, Syafruddin D, Rogers WO: **A model for transmission of partial resistance to anti-malarial drugs**. *Math Biosci Eng* 2009, **6**(3):649-661.

259. White LJ, Maude RJ, Pongtavornpinyo W, Saralamba S, Aguas R, Van Effelterre T, Day NP, White NJ: **The role of simple mathematical models in malaria elimination strategy design**. *Malaria journal* 2009, **8**:212.

260. Kouyos RD, zur Wiesch PA, Bonhoeffer S: **On being the right size: The impact of population size and stochastic effects on the evolution of drug resistance in hospitals and the community**. *PLoS Pathogens* 2011, **7**(4).

261. Tchuenche JM, Chiyaka C, Chan D, Matthews A, Mayer G: **A mathematical model for antimalarial drug resistance**. *Mathematical medicine and biology : a journal of the IMA* 2011, **28**(4):335-355.

262. Maude RJ, Socheat D, Nguon C, Saroth P, Dara P, Li G, Song J, Yeung S, Dondorp AM, Day NP *et al*: **Optimising strategies for Plasmodium falciparum malaria elimination in Cambodia: primaquine, mass drug administration and artemisinin resistance**. *PLoS One* 2012, **7**(5):e37166.

263. Okosun KO, Makinde OD: **On a drug-resistant malaria model with susceptible individuals without access to basic amenities**. *Journal of Biological Physics* 2012, **38**(3):507-530.

264. Agusto FB, Adekunle AI: **Optimal control of a two-strain tuberculosis-HIV/AIDS co-infection model**. *Bio Systems* 2014, **119**:20-44.

265. Maude RJ, Nguon C, Dondorp AM, White LJ, White NJ: **The diminishing returns of atovaquone-proguanil for elimination of Plasmodium falciparum malaria: modelling mass drug administration and treatment**. *Malaria journal* 2014, **13**:380.

266. Mwanga GG, Haario H, Nannyonga BK: **Optimal control of malaria model with drug resistance in presence of parameter uncertainty**. *Applied Mathematical Sciences* 2014(53-56):2701-2730.

267. Ruiz D, Brun C, Connor SJ, Omumbo JA, Lyon B, Thomson MC: **Testing a multi-malaria-model ensemble against 30 years of data in the Kenyan highlands**. *Malaria Journal* 2014, **13**(1).

268. Fatmawati, Tasman H: **An optimal control strategy to reduce the spread of malaria resistance**. *Mathematical biosciences* 2015, **262**:73-79.

269. Forouzannia F, Gumel A: **Dynamics of an age-structured two-strain model for malaria transmission**. *Applied Mathematics and Computation* 2015, **250**:860-886.

270. Legros M, Bonhoeffer S: **A combined within-host and between-hosts modelling framework for the evolution of resistance to antimalarial drugs**. *Journal of the Royal Society, Interface* 2016, **13**(117).

271. Klein EY: **The impact of heterogeneous transmission on the establishment and spread of antimalarial drug resistance**. *Journal of Theoretical Biology* 2014, **340**:177-185.

272. Flegg JA, Patil AP, Venkatesan M, Roper C, Naidoo I, Hay SI, Sibley CH, Guerin PJ: **Spatiotemporal mathematical modelling of mutations of the dhps gene in African Plasmodium falciparum**. *Malaria journal* 2013, **12**:249.

273. Alexander N, Sutherland C, Roper C, Cissé B, Schellenberg D: **Modelling the impact of intermittent preventive treatment for malaria on selection pressure for drug resistance**. *Malaria Journal* 2007, **6**.
